# Adverse Experiences in Brief Meditation Practices: Randomized Controlled Trial

**DOI:** 10.2196/95628

**Published:** 2026-09-14

**Authors:** Otto Simonsson, Zishan Jiwani, Mini Ruiz, Jayanth Narayanan, Walter Osika, Matthew J Hirshberg, Simon B Goldberg

**Affiliations:** 1 Karolinska Institutet Solna, Stockholm Sweden; 2 University of Wisconsin–Madison Madison, WI United States; 3 Northeastern University Boston, MA United States

**Keywords:** meditation, mindfulness, gratitude, self-compassion, adverse, machine learning, distressing, negative

## Abstract

**Background:**

Meditation has become increasingly popular in recent decades. However, relatively little remains known about the prevalence of and risk factors for adverse experiences related to a single meditation practice.

**Objective:**

The objective of our study was to examine adverse experiences associated with 3 brief, digitally delivered meditation practices (mindfulness, self-compassion, and gratitude) relative to using the internet as usual, as well as to investigate whether preintervention characteristics could predict such outcomes.

**Methods:**

In a secondary analysis of a randomized controlled trial using samples that were representative of the US and UK adult populations with regard to ethnicity, sex, and age, we examined adverse experiences associated with 3 brief (ie, 5 or 10 minutes) meditation practices (ie, mindfulness, self-compassion, and gratitude) relative to using the internet as usual. We also investigated the potential of using preintervention characteristics to predict such outcomes.

**Results:**

A total of 5049 participants completed all preintervention measures and were randomly assigned to meditation or control conditions. Across the sample, 4.1% (204/4925) of participants reported having a distressing experience during the intervention, and 7.1% (348/4908) of participants experienced an increase in negative affect from before to after the intervention. The results showed that participants who were randomized to a brief meditation intervention were no more likely to report a distressing experience than those who were randomized to use the internet as usual (odds ratio [OR] 1.05, 95% CI 0.76-1.47; *P*=.76). The results also showed that participants who were randomized to a brief meditation intervention were less likely to report clinically relevant increases in negative affect relative to using the internet as usual (OR 0.63, 95% CI 0.50-0.80; *P*<.001). Notably, participants in the 10-minute condition had a significantly higher likelihood of reporting a distressing experience than those in the 5-minute condition (OR 1.42, 95% CI 1.07-1.89; *P*=.02). Preintervention characteristics showed acceptable discrimination ability to predict a distressing experience (area under the curve=0.73) and slightly lower ability to predict increased negative affect (area under the curve=0.67).

**Conclusions:**

Taken together, we found that the brief, digitally delivered meditation practices tested in this study carry risks of adverse experiences that are comparable to or lower than those of typical activities on the internet; 10-minute condition was more likely to result in distressing experiences than 5-minute condition; and adverse responses to a brief meditation practice can, at least to a certain degree, be predicted using preintervention characteristics.

**Trial Registration:**

Open Science Framework 94HKS; https://osf.io/94hks/overview

## Introduction

Meditation has become increasingly popular over the past 2 decades, particularly in the United States and the United Kingdom [1-6]. For example, between 2002 and 2022, past-year use of meditation among American adults increased from 7.8% to 18.3% [1], with millions of individuals using smartphone apps to learn and practice meditation [5-8]. Such widespread adoption of meditation may have public health benefits [9], but there may also be risks associated with these practices (eg, anxiety and depression) [10-19]. It is therefore important to investigate the prevalence of and risk factors for adverse experiences related to meditation.

The studies that have investigated the prevalence of meditation-related adverse experiences have used a variety of designs and sampling approaches [20], each with its own limitations. For instance, in a cross-sectional study with a sample representative of the US adult population with regard to ethnicity, sex, and age, 32.3% of participants with lifetime exposure to meditation reported challenging, difficult, or distressing experiences they attributed to their meditation practice, whereas 50.0% endorsed at least one item on a multi-item checklist of specific adverse experiences [10]. Because the participants were asked about lifetime meditation-related adverse experiences, however, the study could not estimate the prevalence of adverse experiences associated with a specific meditation program. Notably, several recent randomized trials have estimated rates of adverse experiences in specific meditation programs, both in person and via self-guided apps [21-23]. These studies have, however, lacked nationally representative samples and have not directly compared different meditation programs, which limits the ability to generalize results to a broader population and estimate the relative safety of different meditation programs.

Even when there are direct comparisons of different meditation programs, it may not be possible to accurately estimate the risk of adverse experiences associated with a single meditation practice. Meditation programs often include various meditation practices (eg, mindfulness, self-compassion, and gratitude), and comparisons of multicomponent meditation programs limit the ability to identify which specific meditation practices may be associated with adverse experiences. Hence, it would be helpful to compare a single session of different meditation practices—both with each other and with an appropriate control condition—to better understand the absolute and relative risks of engaging in these practices (even once), especially as single meditation inductions are a widely implemented delivery format (eg, in educational and clinical settings) [24,25] and brief guided practices are foundational in both in-person and self-guided app programs. Along with evaluating the effect of different practices, it would be helpful to investigate the effects of specific instructor characteristics (eg, gender and ethnicity) on meditation-related adverse experiences, a factor that could theoretically have an effect on these types of experiences and yet has not been examined in brief, single-session interventions. Because previous research has documented variability in meditation teacher effects on participants’ trajectories through meditation-related challenges [26], as well as therapist effects in psychotherapy more broadly [27], it is entirely plausible that instructor characteristics may impact how individuals react to even brief meditation practices. Taken together, such research would have clinical and practical relevance and could help optimize multicomponent meditation programs by identifying which meditation practices and instructor characteristics are most likely to cause adverse experiences.

Recent research suggests that meditation-related adverse experiences may be more common among certain populations and under certain conditions. For instance, preexisting mental illness and various forms of trauma, as well as meditation app use and meditation retreat attendance, have shown positive associations with meditation-related adverse experiences [10,11,28,29]. These findings suggest that the population, the delivery method, and the length or intensity of meditation practice (ie, biological gradient) [16,30] may be important predictors of meditation-related adverse experiences. However, because individual predictors often provide limited insights on their own, machine learning approaches that analyze multiple predictors simultaneously may be particularly helpful to identify participants at higher risk of meditation-related adverse experiences.

In a secondary analysis of a randomized controlled trial using samples that were representative of the US and UK adult populations with regard to ethnicity, sex, and age [31], we examined adverse experiences associated with 3 brief meditation practices (ie, mindfulness, self-compassion, and gratitude) relative to a control condition in which participants were instructed to use the internet as they typically do—an ecologically valid, real-world comparator for internet-delivered meditation practices. More specifically, we investigated whether the likelihood of adverse experiences varied by group assignment, intervention length, or speaker voice, as well as whether baseline characteristics predicted the likelihood of adverse experiences. Our use of a factorial design [32] allowed us to efficiently test the impact of several factors within a single experiment.

## Methods

### Participants

Data collection for this study took place in 2023 between November and December. The participants (≥18 years) were recruited through Prolific Academic using the platform’s representativeness function (ie, samples were stratified across age, sex, and ethnicity) for the United States and United Kingdom. The study took approximately 25 minutes to complete.

### Ethical Considerations

Study procedures were determined to be exempt from review by the Institutional Review Board at the University of Wisconsin–Madison, and informed consent was obtained digitally from participants. Participants were informed about the study procedures and their rights as research participants. Participant data were fully anonymized as soon as practicable after data collection by removing identifying information. Participants were paid US $3.20 (£2.50) for study completion.

### Procedure

Before randomization, participants completed items assessing various baseline characteristics to provide a comprehensive pool of candidate predictors of adverse experiences (eg, demographic variables, psychological traits, clinical factors, meditation experience, and technology use history). Participants were also presented with an attention check before randomization (“I have been randomly selecting responses on this survey”). After completion of these items, participants were randomized via the randomizer function in the Qualtrics survey platform (Qualtrics International Inc) using a 4 × 2 × 2 factorial randomized trial design that crossed intervention type (mindfulness, self-compassion, gratitude, and internet as usual) by intervention length (5 minutes and 10 minutes) and speaker voice (male and female), yielding 14 unique conditions given that there was no speaker voice type in the control condition (see Table S1 in Multimedia Appendix 1 for a factorial design overview). The sample sizes in the control condition were double the size of those for the meditation conditions to retain the balance necessary for optimal efficiency [32]. Once the participants had completed their allocated intervention, they completed additional items related to their current psychological state, as well as their psychological state during the intervention (see Table S2 in Multimedia Appendix 1 for a list of single survey items and questionnaires).

### Interventions

The participants who were randomized to one of the meditation conditions were presented with a 5- or 10-minute meditation recording narrated by either a male or female voice, which was taken from the Healthy Minds Program app [33]. In the mindfulness condition, participants were instructed to pay attention to what was happening in the present moment with an attitude of nonjudgment; in the self-compassion condition, participants were instructed to generate feelings of kindness and friendliness toward themselves; in the gratitude condition, participants were instructed to generate feelings of gratitude and appreciation. The participants who were randomized to the control condition were instructed to spend 5 or 10 minutes (based on their group assignment) using the internet in ways in which they typically did. To support engagement with the assigned intervention, the study page was configured such that participants could not continue the study until the full duration of the assigned audio recording (or the corresponding 5- or 10-minute internet-as-usual window) had elapsed, providing a minimum time-on-task constraint. Participants also completed a manipulation check immediately after the intervention in which they were asked to indicate what they had been instructed to do, with 4 response options corresponding to the 4 intervention types: “pay attention to what was happening in the present moment,” “generate feelings of kindness,” “generate feelings of gratitude,” and “use the internet in ways you typically do.”

### Measures

We operationalized adverse experiences as (1) reporting having had a distressing experience during the intervention and (2) increased negative affect from before to after the intervention. The first measure was completed after the intervention only and asked the following: “Did you have a distressing experience during the task you just completed?” Previous research has at times operationalized meditation-related adverse experiences as having had challenging, difficult, or distressing experiences as a result of the meditation practice [12,34], but the use of multiple qualifiers (ie, “challenging,” “difficult,” and “distressing”) complicates interpretation and reduces measurement precision—many activities may be challenging or difficult (eg, learning to play the violin or swimming) but would typically not be considered adverse experiences simply for that reason. We therefore asked participants only about *distressing* experiences to increase interpretability, but it should be noted that this restriction reduces direct comparability with studies that have asked more broadly about challenging, difficult, or distressing experiences. The second measure was a clinically relevant increase—defined as a standardized mean difference of 0.24 [35]—in the negative affect subscale of the Positive and Negative Affect Schedule [36] from before to after the intervention. This measure was intended to provide a complementary assessment to the first measure by capturing increases in negative mood.

### Analysis Plan

#### Overview

All analyses were performed using the R statistical software (R Foundation for Statistical Computing) [37], and the *tidyverse* ecosystem of packages [38] was used for data wrangling. Preprocessing and analyses for machine learning models were conducted using the *tidymodels* ecosystem of packages [39]. Given that 2 different operationalizations of adverse experiences were examined, we applied a Bonferroni correction and set statistical significance at *P*<.025 to control for type I error rate [40]. The internet-as-usual condition was coded as the reference group when comparing active conditions to control, and the gratitude condition was coded as the reference group for comparisons among active conditions. For models involving intervention length or speaker voice, the 5-minute condition and female speaker voice served as the reference categories. For the comparison among the 3 active conditions, we fit a logistic regression with intervention condition as a 3-level factor, which provides pairwise contrasts for self-compassion vs gratitude and mindfulness vs gratitude. The third pairwise contrast (self-compassion vs mindfulness) was obtained from the same model using estimated marginal means. Due to the low base rates for adverse experiences, we conducted Firth penalized logistic regression models as sensitivity analyses for the main effect models. Firth penalized logistic regression reduces small-sample and rare-event bias in maximum likelihood estimates by applying a penalty that shrinks coefficients toward 0 and yields finite estimates even in cases of separation [41,42]. In cases in which Firth penalized and standard logistic regression models yielded no meaningful differences, standard model results are reported in the main text for simplicity. While no a priori power analysis was conducted for these secondary outcomes, the study had more than 80% power at an α value of .05 to detect odds ratios (ORs) of 1.55 or larger for distressing experiences (base rate=4.1%) and 1.40 or larger for increased negative affect (base rate=7.1%) at the observed sample size.

Missing data on both outcomes were low (124/5049, 2.5% and 141/5049, 2.8%). We examined whether missingness was associated with key design variables. Missingness did not differ by intervention group, speaker voice, or baseline negative affect (*P*>.13 in all cases) but was higher in the 10-minute condition than the 5-minute condition (88/2524, 3.5% vs 36/2525, 1.4% for distressing experience; 96/2524, 3.8% vs 45/2525, 1.8% for increased negative affect; *P*<.001 in all cases). The magnitude of this length effect was comparable across active and control conditions (approximately 2-fold increase in both), suggesting that elevated missingness in the 10-minute condition reflects task length rather than distress-related dropout. Given that missing data on both outcomes were less than 5%, which is commonly considered ignorable [43], we conducted complete-case analyses (ie, participants with missing data on a given outcome were excluded from analyses involving that outcome).

#### Research Question 1

Our first research question was preregistered as an exploratory analysis whereby we assessed whether the likelihood of adverse experiences varied by group assignment. We conducted 2 sets of analyses. First, we assessed whether adverse experiences varied between active (mindfulness, self-compassion, and gratitude) and control (internet as usual) conditions. We fit 2 single-predictor logistic regression models with distressing experience and increased negative affect as outcomes. Second, we fit 2 additional single-predictor logistic regression models excluding the control condition to compare the likelihood of adverse experiences (distressing experience and increased negative affect) across the 3 active intervention conditions.

#### Research Question 2

Our second research question assessed whether the likelihood of adverse experiences varied by intervention length (5 vs 10 minutes) or speaker voice (male vs female). To analyze variability by intervention length, we fit 2 single-predictor logistic regression models with distressing experience or increased negative affect as outcomes and intervention length as the predictor. We also tested interaction effects between intervention length and group type (active vs control) using logistic regression models with the corresponding interaction term included to determine whether intervention length effects varied by intervention type. Additionally, we examined interactions between intervention length and specific meditation practices (mindfulness, self-compassion, and gratitude) excluding the control condition. For speaker voice analyses, we also excluded the control condition (which did not have a speaker voice component) and conducted similar logistic regression analyses with speaker voice as the single predictor for both outcomes (distressing experience and increased negative affect). Regarding intervention length, we tested interactions between speaker voice and meditation type to assess whether speaker voice effects differed across the 3 active interventions.

#### Research Question 3

Our third research question assessed whether baseline characteristics predicted the likelihood of adverse experiences. To address this question, we used machine learning for 2 main reasons. First, machine learning approaches can handle large sets of correlated predictors, which was useful given our interest in exploring a fairly large number of candidate baseline variables [44]. Second, machine learning is particularly well suited to situations in which prediction (rather than explanation) is the priority [45]. We implemented a nested cross-validation (CV) approach comparing 2 machine learning algorithms: elastic net regularization and random forest. Elastic net was implemented using the *glmnet* package, and random forest was implemented using the *ranger* package, both via the *tidymodels* ecosystem. Elastic net was used because it prevents overfitting by handling correlated predictors and performing feature selection through regularization penalties, whereas random forest was included as a flexible, nonparametric model that may outperform linear models when relationships are nonlinear and is often robust to overfitting [46,47].

For elastic net, we preprocessed the data using median imputation for missing numeric values, mode imputation for missing nominal values, Yeo-Johnson transformations for numeric variables, dummy coding for categorical variables, feature normalization, and removal of 0 variance predictors. Random forest preprocessing was simplified, excluding normalization and transformations as they are unnecessary for tree-based models [48]. Using 10-fold nested CV, we tuned regularization parameters (penalty and mixture) for elastic net and tree parameters (mtry and minimum node size) for random forest to identify optimal configurations for each approach. For elastic net, the penalty was tuned over 50 values on a log scale (e⁻⁸ to e⁸), and mixture was tuned over 6 values (0 to 1). For random forest, mtry was tuned over {10, 20, 30, 40, 50}, and minimum node size was tuned over {5, 10, 20, 30, 40} with 1000 trees. All other settings were left at their defaults. Nested CV uses 2 levels of data splitting: an outer loop that evaluates the final model performance and an inner loop that optimizes model hyperparameters. Model performance was assessed using the area under the curve (AUC) metric for the receiver operating characteristic curve, with values ranging from 0.5 (no better than chance) to 1.0 (perfect discrimination) and values of 0.5 to 0.7 indicating poor discrimination, values of 0.7 to 0.8 indicating acceptable discrimination, values of 0.8 to 0.9 indicating excellent discrimination, and values of 0.9 or higher indicating outstanding discrimination [49].

Finally, if elastic net was selected as the best-performing algorithm, we planned to review and report its standardized parameter estimates (ie, β values) to rank order the magnitude of the effects of its predictors on the outcome. If random forest was the best-performing algorithm, we planned to extract variable importance scores, which quantify the relative contribution of each predictor to the model’s predictive accuracy based on the mean decrease in node impurity across all trees in the forest.

## Results

### Descriptive Statistics

A total of 5260 individuals participated in the study, of whom 155 (2.9%) were excluded because they failed the attention check (“I have been randomly selecting responses on this survey”) and 56 (1.1%) were excluded due to incomplete responses. The remaining 5049 participants completed all preintervention measures and were randomly assigned to mindfulness (n=1262, 25.0%), self-compassion (n=1268, 25.1%), gratitude (n=1260, 25.0%), or control (n=1259, 24.9%; Figure 1). Participants had a mean age of 45.34 (SD 15.58) years, with 50.7% (2558/5049) identifying as female, 80.5% (4062/5049) identifying as White individuals, 84.4% (4261/5049) identifying as heterosexual, and 70.8% (3573/5049) being employed. Of all the meditation types assessed, mindfulness meditation had the highest proportion of participants reporting ever having tried it prior to the study (2542/5049, 50.3%). Across the sample, 4.1% (204/4925) of the participants reported having a distressing experience during the intervention, and 7.1% (348/4908) of the participants experienced an increase in negative affect from before to after the intervention (Table 1; see Table S3 in Multimedia Appendix 1 for more information on adverse experiences). Missing data for the 2 outcomes were minimal, with distressing experience missing for 2.5% (124/5049) of the participants and increased negative affect missing for 2.8% (141/5049) of the participants. As noted above, missingness was less than 5% across the conditions and, thus, considered ignorable (Table S4 in Multimedia Appendix 1).

**Figure 1:**
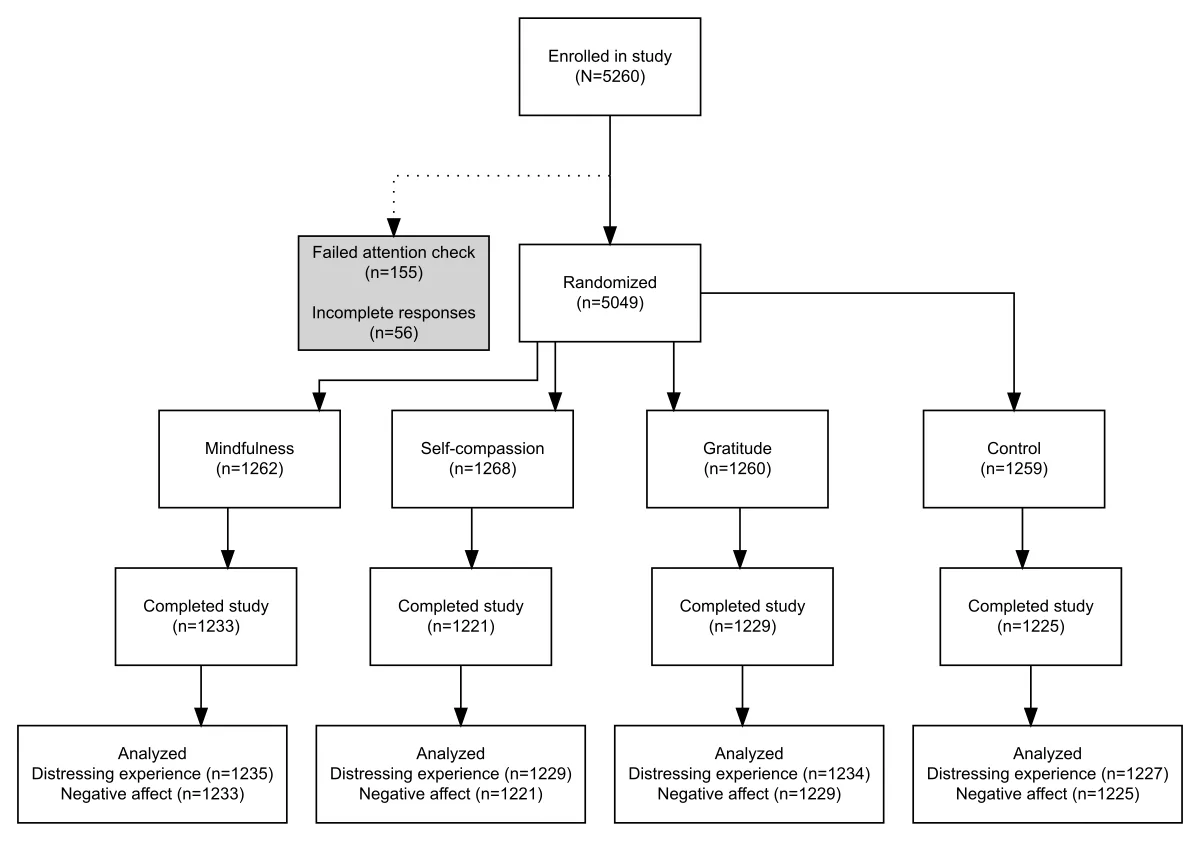
CONSORT (Consolidated Standards of Reporting Trials) flow diagram. “Incomplete responses” refers to participants who did not complete all preintervention (ie, before randomization) survey items. “Completed study” refers to participants who completed the intervention and all pre- and postintervention survey items. Postintervention negative affect was measured after the distressing experience item, explaining the difference in the number of analyzed participants across these 2 measures. Participants were randomized in a 4 × 2 × 2 factorial design (intervention type × length × speaker voice), yielding 14 unique conditions, as there was no speaker voice in the control condition. Allocation is shown here by intervention type, collapsed across length and voice.

**Table 1:** Sample descriptive statistics (N=5049).

Variable			Values^a^		Skewness		Kurtosis
Control, n/N (%)			1259/5049 (24.9)		—^b^		—
Gratitude, n/N (%)			1260/5049 (25.0)		—		—
Self-compassion, n/N (%)			1268/5049 (25.1)		—		—
Mindfulness, n/N (%)			1262/5049 (25.0)		—		—
Intervention length (min), n/N (%)					—		—
	5	2525/5049 (50.0)					
	10	2524/5049 (50.0)					
Speaker voice (male)^c^, n/N (%)			1894/3790 (50.0)		—		—
Distressing experience^d^, n/N (%)			204/4925 (4.1)		—		—
Increased negative affect^e^, n/N (%)			348/4908 (7.1)		—		—
Age (y), mean (SD; range)			45.34 (15.58; 18-100)		0.14		1.91
Discrimination experiences, mean (SD; range)			1.67 (1.02; 1-4)		1.16		2.85
Religiosity^f^, mean (SD; range)			1.99 (1.26; 1-5)		1.06		2.94
DMPI^g^, mean (SD; range)							
	Perceived benefit	2.85 (0.93; 1-5)		0.00		2.42	
	Knowledge barriers	3.21 (1.14; 1-5)		−0.32		2.09	
	Pragmatic barriers	2.25 (0.97; 1-5)		0.72		2.95	
	Cultural barriers	1.66 (0.73; 1-5)		1.38		5.22	
	Total	2.46 (0.64; 1-5)		−0.05		3.03	
Growth mindset, mean (SD; range)			4.61 (1.12; 1-6)		−0.73		2.97
Extraversion, mean (SD; range)			3.47 (1.68; 1-7)		0.3		2.13
Agreeableness, mean (SD; range)			5.34 (1.25; 1-7)		−0.57		2.77
Conscientiousness, mean (SD; range)			5.43 (1.31; 1-7)		−0.78		2.95
Emotional stability, mean (SD; range)			4.72 (1.62; 1-7)		−0.36		2.18
Openness, mean (SD; range)			5.07 (1.27; 1-7)		−0.49		2.79
Lifetime mindfulness practice (h), mean (SD; range)			1.06 (1.33; 0-6)		1.27		4.34
Mindfulness practice (d per wk), mean (SD; range)			0.58 (1.34; 0-7)		2.86		11.36
Lifetime kindness practice (h), mean (SD; range)			0.6 (1.18; 0-6)		2.17		7.55
Kindness practice (d per wk), mean (SD; range)			0.39 (1.19; 0-7)		3.65		16.84
Lifetime gratitude practice (h), mean (SD; range)			0.86 (1.31; 0-6)		1.63		5.33
Gratitude practice (d per wk), mean (SD; range)			0.56 (1.43; 0-7)		3.03		12.04
Loneliness, mean (SD; range)			2.27 (1.17; 1-5)		0.61		2.31
Self-compassion, mean (SD; range)							
	Kindness	3.16 (0.98; 1-5)		−0.14		2.53	
	Common humanity	3.13 (1.02; 1-5)		−0.16		2.44	
	Mindfulness	3.55 (0.92; 1-5)		−0.45		2.9	
	Self-judgment	2.99 (1.17; 1-5)		0.11		2.11	
	Isolation	2.89 (1.17; 1-5)		0.19		2.11	
	Overidentification	2.95 (1.23; 1-5)		0.12		1.95	
	Total	3.11 (0.82; 1-5)		0.01		2.64	
Mindfulness, mean (SD; range)							
	Observe	3.11 (0.89; 1-5)		−0.15		2.69	
	Describe	3.35 (0.95; 1-5)		−0.26		2.6	
	Awareness	3.45 (0.91; 1-5)		−0.08		2.44	
	Nonjudgment	3.63 (1.01; 1-5)		−0.43		2.43	
	Nonreaction	2.98 (0.88; 1-5)		−0.05		2.88	
	Total	3.31 (0.58; 1-5)		0.03		3.15	
Gratitude, mean (SD; range)			5.3 (1.22; 1-7)		−0.78		3.32
Anxiety, mean (SD; range)			2.06 (1.07; 1-5)		0.8		2.62
Depression, mean (SD; range)			2 (1.12; 1-5)		0.91		2.73
Stress, mean (SD; range)			2.62 (0.92; 1-5)		0.24		2.57
Rumination, mean (SD; range)							
	Brooding	2 (0.71; 1-4)		0.45		2.56	
	Reflection	3.32 (0.68; 1-4)		−0.91		3.23	
Negative affect, mean (SD; range)			1.45 (0.7; 1-5)		2.03		7.04
Positive affect, mean (SD; range)			2.95 (0.95; 1-5)		0.09		2.33
Female sex, n/N (%)			2558/5049 (50.7)		—		—
Heterosexual orientation, n/N (%)			4261/5049 (84.4)		—		—
Transgender identity, n/N (%)			77/5049 (1.5)		—		—
In a relationship, n/N (%)			2963/5049 (58.7)		—		—
College education, n/N (%)			2896/5049 (57.4)		—		—
Currently employed, n/N (%)			3573/5049 (70.8)		—		—
Ever tried mindfulness practice, n/N (%)			2542/5049 (50.3)		—		—
Ever tried kindness practice, n/N (%)			1331/5049 (26.4)		—		—
Ever tried gratitude practice, n/N (%)			1986/5049 (39.3)		—		—
Have you ever used the internet to access information to support your mental health?, n/N (%)			3190/5049 (63.2)		—		—
Have you ever used the internet to access treatments or interventions (e.g., teletherapy, self-guided programs such as mindfulness training or cognitive behavioral therapy) to support your mental health?, n/N (%)			1867/5049 (37.0)		—		—
Internet mental health forums, n/N (%)			1521/5049 (30.1)		—		—
Internet-based CBT^h^, n/N (%)			725/5049 (14.4)		—		—
Have you ever used social media to access information to support your mental health?, n/N (%)			1807/5049 (35.8)		—		—
Have you ever used social media to access treatments or interventions (e.g., teletherapy, self-guided programs such as mindfulness training or cognitive behavioral therapy) to support your mental health?, n/N (%)			684/5049 (13.5)		—		—
Social media mental health forums, n/N (%)			1130/5049 (22.4)		—		—
Have you ever downloaded an app for your mental health?, n/N (%)			1949/5049 (38.6)		—		—
Have you ever used a smartphone app to access information to support your mental health?, n/N (%)			1803/5049 (35.7)		—		—
Have you ever used a smartphone app to access treatments or interventions (e.g., teletherapy, self-guided programs such as mindfulness training or cognitive behavioral therapy) to support your mental health?, n/N (%)			1171/5049 (23.2)		—		—
Have you ever used ChatGPT or similar software based on artificial intelligence to support your mental health?, n/N (%)			516/5049 (10.2)		—		—
Internet access, n/N (%)			5038/5049 (99.8)		—		—
Race or ethnicity, n/N (%)					—		—
	Asian	314/5049 (6.2)					
	Black	417/5049 (8.3)					
	Mixed	101/5049 (2.0)					
	White	4062/5049 (80.5)					
	Other	155/5049 (3.1)					

^a^For all self-reported scales, the observed ranges are identical to possible ranges.

^b^Not applicable.

^c^Missing: 24.9% (1259/5049). The control group did not have a speaker voice.

^d^Missing: 2.5% (124/5049).

^e^Missing: 2.8% (141/5049).

^f^Religiosity was assessed using the question “How religious are you?” with responses scored from 1 (not at all religious) to 5 (very religious).

^g^DMPI: Determinants of Meditation Practice Inventory–Revised.

^h^CBT: cognitive behavioral therapy.

### Research Question 1: Group Differences in Adverse Experiences

When comparing the active interventions (mindfulness, self-compassion, and gratitude) to the control condition (internet as usual), we observed no difference in the likelihood of reporting a distressing experience (OR 1.05, 95% CI 0.76-1.47; *P*=.76; Table 2). We observed that participants in the 3 active intervention conditions had a significantly lower likelihood of increased negative affect than those in the control condition (OR 0.63, 95% CI 0.50-0.80; *P*<.001; Table 2). The 3 meditation conditions did not differ from each other in the likelihood of either reporting a distressing experience or experiencing increased negative affect (*P*>.20 in all cases; Table 2). Results from the Firth penalized models were consistent with the standard logistic regressions, with no meaningful differences in the main effects (Table S5 in Multimedia Appendix 1).

**Table 2:** Examining the likelihood of adverse experiences by group assignment^a^.

Model	Outcome	Comparison	OR^b^ (95% CI)	*z* score (*df*)	*P* value
Active vs control	Distressing experience	Active vs control	1.05 (0.76-1.47)	0.3 (4923)	.76
Active vs control	Increased negative affect	Active vs control	0.63 (0.50-0.80)	−3.85 (4906)	<.001
Among active conditions	Distressing experience	Self-compassion vs gratitude	0.77 (0.52-1.15)	−1.27 (3695)	.20
Among active conditions	Distressing experience	Mindfulness vs gratitude	0.84 (0.57-1.23)	−0.89 (3695)	.38
Among active conditions	Increased negative affect	Self-compassion vs gratitude	0.87 (0.62-1.20)	−0.87 (3680)	.38
Among active conditions	Increased negative affect	Mindfulness vs gratitude	0.91 (0.66-1.25)	−0.59 (3680)	.55

^a^Groups listed second in the comparison column were coded as the reference group.

^b^OR: odds ratio.

### Research Question 2: Intervention Length and Speaker Voice

When examining intervention length, participants in the 10-minute condition had a significantly higher likelihood of reporting a distressing experience than those in the 5-minute condition (OR 1.42, 95% CI 1.07-1.89; *P*=.02; Table 3). The interaction between intervention length and intervention type (active vs control), as well as the interaction between intervention length and the 3 active intervention conditions, were nonsignificant (*P*>.13 in all cases).

**Table 3:** Examining the likelihood of adverse experiences by intervention length and speaker voice^a^.

Model	Outcome	Model type	Comparison	OR^b^ (95% CI)	*z* score (*df*)	*P* value
Intervention length	Distressing experience	Main effect	10 vs 5 min	1.42 (1.07-1.89)	2.43 (4923)	.02
Intervention length	Increased negative affect	Main effect	10 vs 5 min	1.00 (0.8-1.24)	−0.02 (4906)	.99
Speaker voice	Distressing experience	Main effect	Male vs female voice	0.94 (0.68-1.3)	−0.36 (3696)	.72
Speaker voice	Increased negative affect	Main effect	Male vs female voice	0.86 (0.66-1.13)	−1.08 (3681)	.28

^a^Groups listed second in the comparison column were coded as the reference group.

^b^OR: odds ratio.

Intervention length did not impact likelihood of increased negative affect (OR 1.00, 95% CI 0.8-1.24; *P*=.99). However, there was a significant positive interaction between the 10-minute intervention length and active condition (OR 1.75, 95% CI 1.09-2.8; *P*=.02; Table S6 in Multimedia Appendix 1). As shown in Figure 2, the interaction was driven by lower odds of increased negative affect in the 10-minute versus 5-minute intervention-length condition in the control group, while the odds remained relatively stable across intervention lengths in the active conditions. This directionality of effects was observed when examining each group separately. This analysis showed that, in the control group, 10-minute sessions had a lower likelihood of increased negative affect than 5-minute sessions (OR 0.69, 95% CI 0.46-1.01; *P*=.06), whereas in the active condition, 10-minute sessions had a higher likelihood of increased negative affect than 5-minute sessions (OR 1.20, 95% CI 0.92-1.57; *P*=.19), although neither was significant. The interactions between intervention length and the 3 active intervention conditions were nonsignificant (*P*>.04 in all cases; Table S6 in Multimedia Appendix 1).

**Figure 2:**
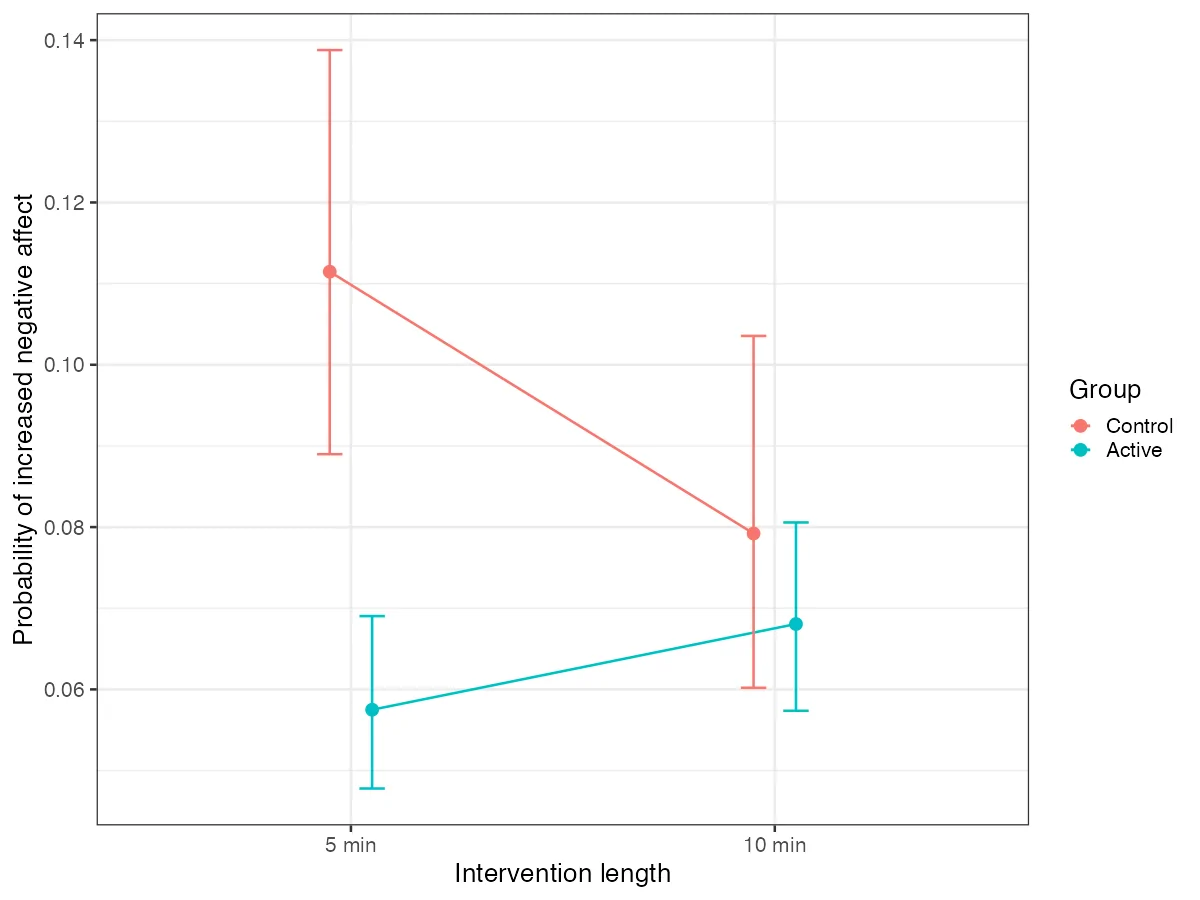
Interaction between intervention length and group assignment (active vs control) in predicting increased negative affect. “Control” refers to internet as usual, and “active” refers to the mindfulness, self-compassion, and gratitude conditions.

Finally, no significant differences were found in the likelihood of adverse experiences based on speaker voice characteristics for either distressing experience or increased negative affect (Table 3). Results from the Firth penalized models were consistent with the standard logistic regressions, with no meaningful differences in the main effects (Table S7 in Multimedia Appendix 1).

### Research Question 3: Predicting Adverse Experiences

Finally, we compared elastic net regularization and random forest algorithms using nested CV for both adverse experience outcomes. Elastic net (AUC=0.73 and 0.67) outperformed random forest (AUC=0.71 and 0.65) for both adverse experience outcomes (ie, distressing experiences and increased negative affect, respectively). As such, we used elastic net as our primary algorithm to interpret performance and assess feature importance. For distressing experience, our model achieved acceptable predictive performance with an AUC of 0.73, indicating acceptable discrimination ability for this previously unexplored prediction problem. Performance was slightly lower for predicting increased negative affect (AUC=0.67), falling in the poor discrimination range. Feature importance analysis revealed that, for distressing experiences, the most important predictors were negative emotional states or traits (state negative affect, anxiety, and depression) and marginalized identities (transgender identity, discrimination experiences, and Black race). In contrast, for increased negative affect, protective factors emerged as key predictors, such as gratitude and nonjudging of experience, as well as group assignment (mindfulness, self-compassion), showing the strongest negative associations with adverse outcomes (see Figures 3 and 4 for the 20 most important variables for distressing experiences and increased negative affect, respectively; see Table S8 in Multimedia Appendix 1 for the complete list of variables with standardized regression coefficients).

**Figure 3:**
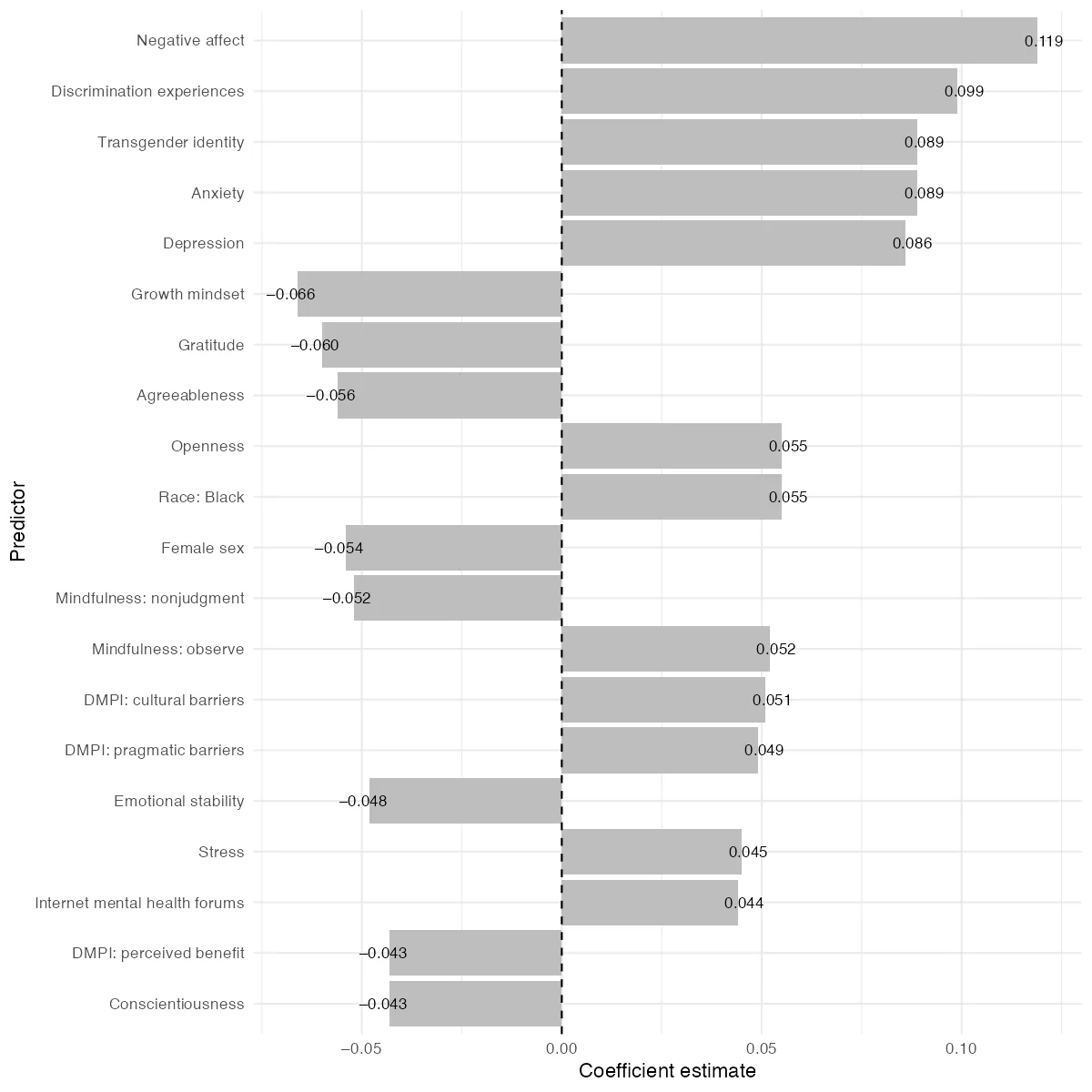
Most important variables in predicting distressing experiences organized by standardized coefficient values. DMPI: Determinants of Meditation Practice Inventory–Revised.

**Figure 4:**
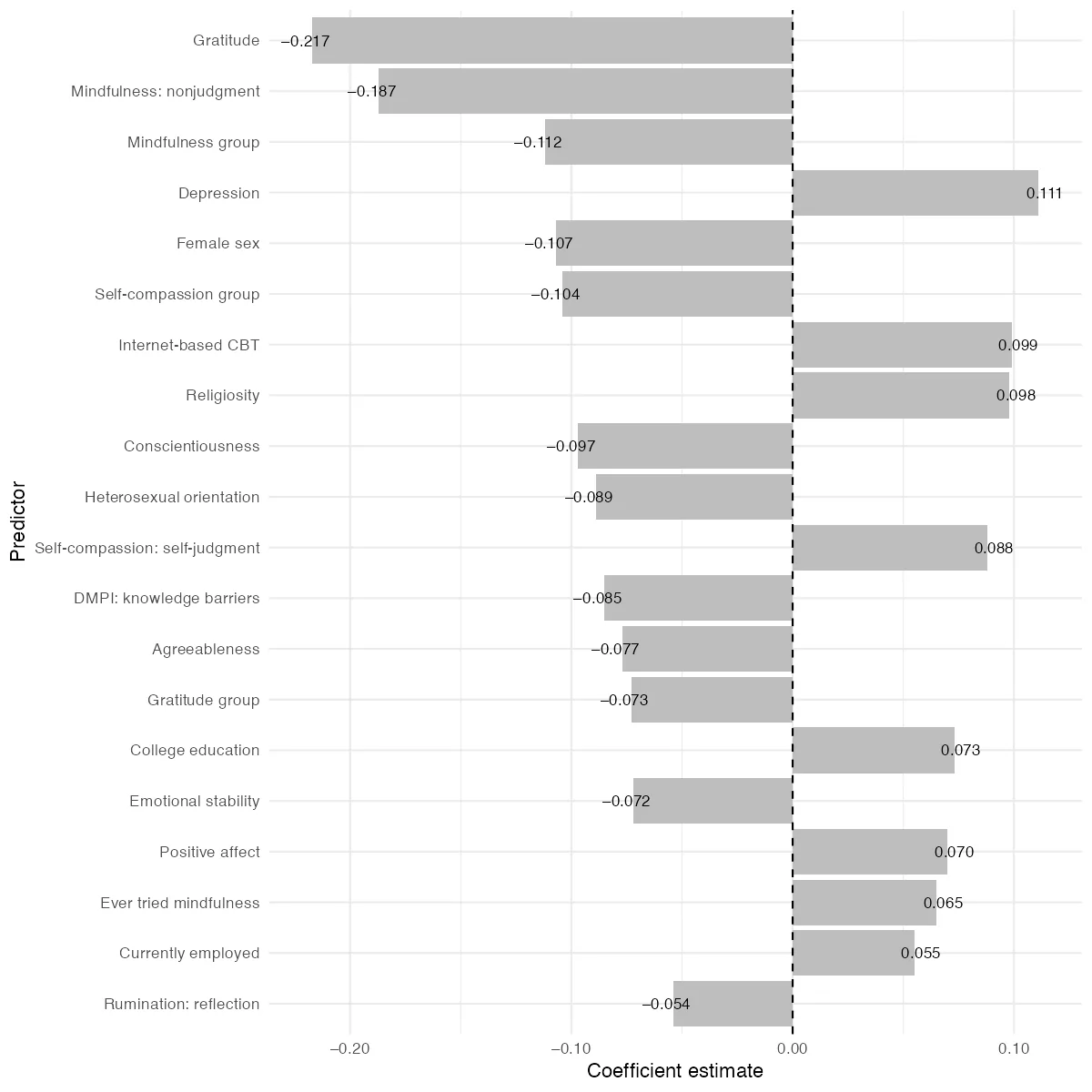
Most important variables in predicting increased negative affect organized by standardized coefficient values. CBT: cognitive behavioral therapy; DMPI: Determinants of Meditation Practice Inventory–Revised.

## Discussion

This study investigated adverse experiences in brief meditation practices using large, representative samples of the US and UK adult populations with regard to ethnicity, sex, and age. Notably, the results showed that participants who were randomized to a brief meditation intervention were no more likely to report a distressing experience than those who were randomized to use the internet as usual. The results also showed that participants who were randomized to a brief meditation intervention were less likely to report clinically relevant increases in negative affect relative to using the internet as usual. Consistent with prior randomized trials that have estimated rates of adverse experiences in meditation programs [21-23,50], our findings suggest that the brief, digitally delivered meditation practices tested in this study carry risks of adverse experiences that are comparable to or lower than those of typical activities on the internet. It underscores the potential for widespread implementation of these interventions as safe approaches to improve well-being.

Further analyses examined whether the likelihood of adverse experiences (distressing experiences and increased negative affect) varied based on aspects of the interventions (intervention length and speaker voice). The results showed that participants randomized to a 10-minute condition were more likely to report a distressing experience than participants randomized to a 5-minute condition. However, given the limited prior meditation experience reported by the sample, it is possible that these findings do not generalize to groups with more meditation experience. It is also possible that these findings are specific to comparison between 5- and 10-minute conditions and may not generalize to longer meditation durations.

Using elastic net regularization as the best-performing machine learning algorithm, preintervention characteristics showed acceptable discrimination ability to predict a distressing experience and slightly lower ability to predict increased negative affect. Notably, negative emotional states or traits (eg, negative affect, anxiety, and depression) and marginalized identities (eg, transgender identity and Black race) emerged as prominent predictors of a distressing experience, whereas positive emotional traits (eg, gratitude and nonjudgment of the experience) as well as group assignment (mindfulness and self-compassion) emerged as protective factors against increased negative affect. The results on emotional states and traits build on prior research that has identified psychological risk factors such as rumination as predictors of response to a digital meditation program [51,52], which highlights the potential of personalized meditation approaches to minimize risks and maximize benefits. The identification of marginalized identities as predictors of distress further extends prior concerns that meditation-based interventions may carry uneven risk profiles across populations, particularly when identity-related stressors and structural inequities are not properly considered [53,54]. These findings should be considered when meditation practices are recommended in cultural contexts shaped by structural inequities, especially as individuals navigating identity-related stressors may be more likely to experience distress during certain meditation practices.

Despite the study’s strengths, it is important to also consider its limitations when interpreting the findings. First, the measures of adverse experiences were self-reported, which has known biases (eg, social desirability) [55]. In particular, the distressing experience outcome was assessed using a single item, which may be less reliable than psychometrically validated self-report checklists for capturing the range and severity of adverse experiences. Second, given that different measurement approaches may yield systematically different prevalence estimates of meditation-related adverse experiences, it is possible that the prevalence estimates observed in this study could represent lower bounds of the true rate of adverse experiences associated with these brief meditation practices. Third, the sample was stratified to be representative of the US and UK national populations with respect to age, sex, and ethnicity, but it may not have been representative regarding other sociodemographic variables. Fourth, participants in the control condition were instructed to use the internet as they typically would, but the way in which they used the internet was neither systematically assessed nor objectively monitored. It is therefore plausible that at least some participants spent their allocated 5- or 10-minute window engaging with emotionally activating material (eg, news coverage and social media), any of which may itself have elicited distressing experiences or increases in negative affect. Fifth, the data-driven approach to identify predictors of adverse experiences was not externally validated in an independent sample. Sixth, this study only examined distressing experiences during the intervention or immediate increases in negative affect after the intervention. The duration of any effects, therefore, remains unknown. Seventh, while we used an attention check to screen out careless responders, we did not implement additional approaches to detect careless response patterns or lack of variability in the data [56], which may have allowed some careless responses to remain in the analytic sample. Future randomized controlled trials with more broadly representative samples, more objective and behavioral measures, more rigorous methodological safeguards, and a longer follow-up should aim to replicate these findings and externally validate data-driven approaches.

In conclusion, this study investigated adverse experiences in brief meditation practices and the potential of using preintervention characteristics to predict such outcomes. Taken together, the findings suggest that the brief, digitally delivered meditation practices tested in this study carry risks of adverse experiences that are comparable to or lower than those of typical activities on the internet; a higher single dose may increase the incidence of distressing experiences; and adverse responses to these meditation practices can, at least to a certain degree, be predicted using preintervention characteristics.

## Appendix

Multimedia Appendix 1 Supplementary tables containing additional study information and analyses.

Multimedia Appendix 2 CONSORT-EHEALTH checklist (V 1.6.1).

## Abbreviations

AUC: area under the curve
CV: cross-validation
OR: odds ratio
