# Supplementary material for: Adverse Experiences in Brief Meditation Practices: Randomized Controlled Trial
Source: JMIR Ment Health. 2026 Sep 14;13:e95628. doi: 10.2196/95628 (PMC13693860; doi:10.2196/95628)
Supplement: Supplementary file 1 [file mental_v13i1e95628_app2.pdf]

CONSORT-EHEALTH (V 1.6.1) – Responses

Manuscript: Adverse experiences in brief meditation practices: a randomized controlled trial

Administrative / Metadata

Your name

Otto Simonsson

Primary Affiliation (short), City, Country

Karolinska Institutet, Solna, Sweden

Your e-mail address

otto.simonsson@ki.se

Title of your manuscript

Adverse experiences in brief meditation practices: randomized controlled trial

Name of your App/Software/Intervention

Meditations from the Healthy Minds Program app

Evaluated Version

Recordings drawn from the Healthy Minds Program app, accessed in 2023 (no specific version number was recorded).

Language(s)

English

URL of your Intervention Website or App

humin.org/wellbeing-tools/app

URL of an image/screenshot (optional)

N/A

Accessibility

Access is free and open (the Healthy Minds Program app is freely available; the specific recordings used in this study were embedded within the Qualtrics survey for trial participants only).

Primary Medical Indication/Disease/Condition

Adverse experiences associated with brief meditation practice in nationally representative samples (US and UK adult populations stratified by age, sex, and ethnicity); not a clinical disease indication.

Primary Outcomes measured in trial

Adverse experiences, operationalized as (1) self-reported distressing experience during the intervention, and (2) clinically relevant increase in negative affect (PANAS) from pre- to post-intervention.

Secondary/other outcomes

Predictors of adverse experiences derived from baseline characteristics (demographics, psychological traits, clinical factors, meditation experience, technology use).

Recommended "Dose"

Single-Session Intervention (a single 5- or 10-minute meditation practice).

Approx. Percentage of Users still using the app as recommended after 3 months

Not applicable – this was a single-session intervention with no follow-up use period (the trial ended after the post-intervention assessment).

Overall, was the app/intervention effective?

Partly – relative to the internet-as-usual control, the brief meditation interventions did not increase the likelihood of a distressing experience and significantly reduced the likelihood of a clinically relevant increase in negative affect. However, ten-minute (vs five-minute) meditation practices were more likely to elicit a distressing experience.

Article Preparation Status/Stage

Accepted.

Journal

JMIR Mental Health

Is this a full powered effectiveness trial or a pilot/feasibility trial?

Fully powered (the parent trial was fully powered; the present analyses are secondary, with >80% power at α=.05 to detect ORs ≥1.55 for distressing experience and ≥1.40 for increased negative affect at the observed sample size).

Manuscript tracking number

95628

Title and Abstract

1a) Identification as a randomized trial in the title

Yes. The full title is: "Adverse experiences in brief meditation practices: randomized controlled trial."

1a-i) Identify the mode of delivery in the title

Rating: 4/5

Partially addressed. The title does not explicitly use the words "web-based," "mobile," or "digital," but the abstract and introduction make the mode of delivery clear: "three brief, digitally-delivered meditation practices (mindfulness, self-compassion, and gratitude) relative to using the internet as usual." The interventions consisted of audio recordings drawn from the Healthy Minds Program app and were delivered through a Qualtrics survey.

1a-ii) Non-web-based components or important co-interventions in title

Rating: 3/5

N/A. There were no non-web-based components or co-interventions; participants completed all study activities online (recruitment, consent, baseline measures, randomization, intervention, and post-intervention assessment).

1a-iii) Primary condition or target group in the title

N/A. The trial was conducted in nationally representative samples of the general adult populations of the US and UK (stratified by age, sex, and ethnicity) and not directed at a specific clinical condition or patient group; the title therefore does not name a primary condition. The target group (general adult population) and the focus on adverse experiences in brief meditation are clearly stated in the abstract.

1b-i) Key features of the intervention and comparator in METHODS of ABSTRACT

Rating: 5/5

Yes. From the abstract: "In a secondary analysis of a randomized controlled trial using samples that were representative of the US and UK adult populations with regards to ethnicity, sex, and age, we examined adverse experiences associated with three brief (i.e., five or ten minutes) meditation practices (i.e., mindfulness, self-compassion, and gratitude) relative to using the internet as usual."

1b-ii) Level of human involvement in METHODS of ABSTRACT

Rating: 4/5

Partially. The abstract describes the interventions as "brief (i.e., five or ten minutes) meditation practices." The Methods section of the main text clarifies that the practices were fully self-guided audio recordings (no live therapist or human contact during the intervention) drawn from the Healthy Minds Program app: "a five- or ten-minute meditation recording, which was taken from the Healthy Minds Program app."

1b-iii) Open vs. closed, web-based vs. face-to-face assessments in METHODS of ABSTRACT

Rating: 4/5

Partially. The abstract specifies an online RCT design with web-based self-assessment. The main text Methods clarify: "The participants (≥18 years old) were recruited through Prolific Academic" and that all data were collected via the Qualtrics survey platform (closed/closed-recruitment online trial; outcomes self-assessed via online questionnaires).

1b-iv) RESULTS section in abstract must contain use data

Rating: 4/5

Yes. From the abstract: "A total of 5,049 participants completed all pre-intervention measures and were randomly assigned to meditation or control conditions. Across the sample, 4.1% of participants reported having a distressing experience during the intervention and 7.1% of participants experienced an increase in negative affect from before to after the intervention." Because the intervention was a single 5- or 10-minute session and the study page enforced a minimum time-on-task (participants could not progress until the full audio had elapsed), the relevant adherence/exposure metric is completion of the assigned session, which is reflected in the analytic sample sizes.

1b-v) CONCLUSIONS/DISCUSSION in abstract for negative trials

Rating: 4/5

Yes. The abstract Conclusions section provides a balanced interpretation: "the brief, digitally-delivered meditation practices tested in this study carry comparable or lower risk of adverse experiences than typical activities on the internet; ten-minute meditation practices were more likely to result in

distressing experiences than five-minute meditation practices; and adverse responses to a brief meditation practice can, at least to a certain degree, be predicted using pre-interventions characteristics."

Introduction

2a-i) Problem and the type of system/solution

Rating: 4/5

Yes. From the Introduction: "Meditation has become increasingly popular over the past two decades, particularly in the United States (US) and the United Kingdom (UK). For example, between 2002 and 2022, past-year use of meditation among US adults increased from 7.8% to 18.3%, with millions of individuals using smartphone applications to learn and to practice meditation. Such widespread adoption of meditation may have public health benefits, but there may also be risks associated with these practices (e.g., anxiety, depression). It is therefore important to investigate the prevalence of and risk factors for adverse experiences related to meditation." The interventions are stand-alone, brief, digitally-delivered meditation practices for the general adult population.

2a-ii) Scientific background and rationale: what is known about the (type of) system

Rating: 4/5

Yes. The Introduction summarizes prior cross-sectional and randomized work on meditation-related adverse experiences and their limitations, e.g.: "in a cross-sectional study with a sample representative of the US adult population…32.3% of participants with lifetime exposure to meditation reported challenging, difficult, or distressing experiences they attributed to their meditation practice, while 50.0% endorsed at least one item on a multi-item checklist of specific adverse experiences." The rationale for using internet-as-usual as the comparator is also explicit: "a control condition in which participants were instructed to use the internet as they typically do – an ecologically valid, real-world comparator for internet-delivered meditation practices."

2b) Specific objectives or hypotheses

Yes. From the Introduction: "In a secondary analysis of a randomized controlled trial using samples that were representative of the US and UK adult populations with regards to ethnicity, sex, and age, we examined adverse experiences associated with three brief meditation practices (i.e., mindfulness, self-compassion, and gratitude) relative to a control condition in which participants were instructed to use the internet as they typically do… More specifically, we investigated whether the likelihood of adverse experiences varied by group assignment, intervention length, or speaker voice, as well as whether baseline characteristics predicted the likelihood of adverse experiences." The three research questions are described in detail in the Analysis Plan.

Methods

3a) Description of trial design (such as parallel, factorial) including allocation ratio

Yes. From the Methods: "participants were randomized via the randomizer function in the Qualtrics survey platform using a 4 x 2 x 2 factorial randomized trial design that crossed intervention type (mindfulness, self-compassion, gratitude, internet-as-usual) by intervention length (five minutes, ten minutes) and by speaker voice (male, female), with 14 unique conditions given that there was no speaker voice type in the control condition. The sample sizes in the control condition were double the size of the meditation conditions to retain balance necessary for optimal efficiency."

3b) Important changes to methods after trial commencement (such as eligibility criteria), with reasons

No important changes to methods were made after trial commencement. The present manuscript reports a secondary analysis with the analytic plan preregistered on OSF (https://osf.io/94hks). Research Question 1 was preregistered as an exploratory analysis; Research Questions 2 and 3 were planned but not preregistered.

3b-i) Bug fixes, Downtimes, Content Changes

Rating: 3/5

No bug fixes, downtimes, content changes, or other unexpected events affecting the intervention or comparator occurred during the trial. The audio recordings were embedded as static media within the Qualtrics survey, and the survey was open to recruitment for a defined window in November–December 2023.

4a) Eligibility criteria for participants

Yes. From the Methods: "The participants (≥18 years old) were recruited through Prolific Academic, using the platform's representativeness function (i.e., samples were stratified across age, sex, and ethnicity) for the US and the UK." No further exclusion criteria beyond age and platform-level eligibility were applied at recruitment; 155 participants (2.9%) were excluded post-hoc for failing the attention check and 56 (1.1%) for incomplete responses.

4a-i) Computer / Internet literacy

Rating: 3/5

Computer/internet literacy was an implicit eligibility criterion: participants were active members of the Prolific Academic online research panel and completed all study procedures (recruitment, consent, baseline questionnaires, randomized intervention, and post-intervention measures) online. This is acknowledged in the Methods ("recruited through Prolific Academic") and is consistent with internet-based RCTs in this field.

4a-ii) Open vs. closed, web-based vs. face-to-face assessments

Rating: 4/5

This was a closed (recruitment-based), purely web-based trial with no face-to-face components. From the Methods: "The participants (≥18 years old) were recruited through Prolific Academic." Prolific assigns a unique participant ID to each registered user and prevented duplicate participation in the study, addressing the risk of multiple identities. Outcomes were self-assessed online through Qualtrics.

4a-iii) Information giving during recruitment

Rating: 3/5

Recruitment materials on Prolific Academic indicated that the study was a research project conducted by the University of Wisconsin-Madison and that it would take approximately 25 minutes to complete, with payment of 3.2 USD (2.5 GBP). The full purpose (including a focus on adverse experiences) was not emphasized in recruitment materials so as not to bias self-selection or expectancies. Informed consent was obtained digitally before any study procedures: "informed consent was obtained digitally from participants."

4b) Settings and locations where the data were collected

Yes. Data were collected entirely online via the Qualtrics survey platform, with participants recruited through Prolific Academic from the US and UK. Data collection took place in November–December 2023.

Study procedures were administered remotely; participants completed the study from their own location at a time of their choosing.

4b-i) Report if outcomes were (self-)assessed through online questionnaires

Rating: 5/5

Yes. All outcomes were self-assessed through online questionnaires delivered via Qualtrics. The distressing-experience item was administered immediately post-intervention ("Did you have a distressing experience during the task you just completed?"), and the negative affect outcome was derived from the PANAS administered both pre- and post-intervention.

4b-ii) Report how institutional affiliations are displayed

Rating: 2/5

The study was hosted on Prolific Academic and Qualtrics. The University of Wisconsin-Madison was identified as the responsible institution in the digital informed consent form, but institutional branding was not prominently featured during the intervention or assessment phases, minimizing potential expectancy effects from institutional affiliation.

5-i) Mention names, credential, affiliations of the developers, sponsors, and owners

Rating: 4/5

The audio recordings were drawn from the Healthy Minds Program app, developed by Healthy Minds Innovations, Inc., a non-profit affiliated with the Center for Healthy Minds at the University of Wisconsin-Madison. Two co-authors (S.B.G. and Z.J.) are affiliated with the Center for Healthy Minds, and one author (M.J.H.) was previously affiliated; this relationship is reflected in the author affiliations and disclosed in the Conflicts of Interest statement.

5-ii) Describe the history/development process

Rating: 3/5

The Healthy Minds Program app and its meditation content have been developed by Healthy Minds Innovations and the Center for Healthy Minds, University of Wisconsin-Madison, and have been evaluated in multiple prior trials. The specific recordings used here were existing app content that had been developed and refined for use in the app prior to this study; no new content was created for the trial.

5-iii) Revisions and updating

Rating: 3/5

The audio recordings used in the trial were embedded as static media within the Qualtrics survey from the start of recruitment (November 2023) until the end of data collection (December 2023). Content was therefore "frozen" for the duration of the trial; no revisions or updates were made to the recordings while the trial was active.

5-iv) Quality assurance methods

Rating: 3/5

The recordings were drawn from the Healthy Minds Program app, which has undergone iterative development and prior evaluation by the Center for Healthy Minds. The study page was configured so that participants could not advance until the full duration of the assigned audio (or the corresponding 5- or 10-minute internet-as-usual window) had elapsed, ensuring minimum exposure to the assigned

condition. A manipulation check was administered immediately after the intervention to verify that participants had received the intended instructions.

5-v) Ensure replicability by publishing the source code, screenshots, screen-capture video, or flowcharts

Rating: 4/5

The study materials, analysis plan, and data are openly available on the Open Science Framework (https://osf.io/94hks/overview). The factorial design is summarized in Supplemental Table 1 and the full list of survey items and questionnaires is provided in Supplemental Table 2. The Healthy Minds Program app, from which the recordings were drawn, is freely available at humin.org/wellbeing-tools/app.

5-vi) Digital preservation

Rating: 3/5

The intervention URL (humin.org/wellbeing-tools/app) is provided. The OSF preregistration and study materials (https://osf.io/94hks) are archived through the Open Science Framework, which provides long-term preservation.

5-vii) Access

Rating: 4/5

Participants accessed the meditation recordings within the Qualtrics survey, on their own device and at a location of their choosing, after being randomized. Participants did not pay to access the intervention; they were paid 3.2 USD (2.5 GBP) for completing the study. Participants did not need an account with the Healthy Minds Program app to take part. The Healthy Minds Program app itself is freely available to the public.

5-viii) Mode of delivery, features/functionalities/components, and theoretical framework

Rating: 5/5

From the Methods: "The participants who were randomized to one of the meditation conditions were presented with a five- or ten-minute meditation recording, which was taken from the Healthy Minds Program app. In the mindfulness condition, participants were instructed to pay attention to what was happening in the present moment with an attitude of non-judgment; in the self-compassion condition, participants were instructed to generate feelings of kindness and friendliness toward themselves; in the gratitude condition, participants were instructed to generate feelings of gratitude and appreciation. The participants who were randomized to the control condition were instructed to spend five or ten minutes (based on their group assignment) using the internet in ways that they typically do." Each meditation condition draws on a distinct theoretical/contemplative tradition (mindfulness/Buddhist meditation; loving-kindness/self-compassion; gratitude practice from positive psychology), with the Healthy Minds Program app's framework rooted in four pillars (awareness, connection, insight, purpose).

5-ix) Describe use parameters

Rating: 4/5

The intervention was a single fixed-duration session (5 or 10 minutes, per random assignment). "To support engagement with the assigned intervention, the study page was configured such that participants could not continue the study until the full duration of the assigned audio recording (or the corresponding five- or ten-minute internet-as-usual window) had elapsed, providing a minimum time-on-task constraint." No additional sessions, ad libitum use, or post-trial dosing were prescribed.

5-x) Clarify the level of human involvement

Rating: 4/5

The interventions were fully automated, self-guided audio meditations with no human contact during the intervention or assessment. There was no live therapist, instructor, technical assistant, or care provider involved in delivering the intervention. The audio recordings were narrated by male or female speakers (the speaker voice factor of the design).

5-xi) Report any prompts/reminders used

Rating: 3/5

This was a single-session, single-sitting trial, so prompts or reminders to return to the intervention were not relevant. Standard within-survey prompts (e.g., the requirement that the audio play in full before participants could proceed) ensured exposure to the intervention; no SMS, email, or telephone prompts were used.

5-xii) Describe any co-interventions (incl. training/support)

Rating: 3/5

There were no co-interventions, training sessions, or human support provided alongside the meditation recordings. Participants received only the assigned audio recording (or the corresponding internet-as-usual window for control).

6a) Completely defined pre-specified primary and secondary outcome measures

Yes. From the Methods: "We operationalized adverse experiences as: (1) reporting having had a distressing experience during the intervention; and (2) increased negative affect from pre- to post-intervention. The first measure was completed post-intervention only and asked: 'Did you have a distressing experience during the task you just completed?'… The second measure was a clinically relevant increase – defined as a standardized mean difference of 0.24 – in the negative affect subscale of the Positive and Negative Affect Schedule (PANAS) from pre-intervention to post-intervention." Baseline characteristics used in the prediction analyses are described in the Procedure section and listed in Supplemental Table 2.

6a-i) Online questionnaires: validation and CHERRIES items

Rating: 4/5

Outcomes were assessed via Qualtrics. The PANAS is a widely used, psychometrically established measure of state affect that has been used in numerous online studies. The single distressing-experience item was developed for this study; this is acknowledged as a limitation: "the distressing experience outcome was assessed with a single item, which may be less reliable than psychometrically validated self-report checklists for capturing the range and severity of adverse experiences." Survey items were presented one screen at a time, with no completeness checks beyond a single attention check, and participants could review/change responses within a page before progressing.

6a-ii) Describe whether and how "use" was defined/measured/monitored

Rating: 4/5

"Use" of the assigned condition was defined as completing the full 5- or 10-minute audio recording (or 5/10-minute internet-as-usual window). The Qualtrics survey enforced a minimum time-on-task constraint by preventing participants from advancing until the full duration had elapsed. A post-intervention manipulation check (asking participants to indicate which instruction they had received) was used to verify that participants attended to the assigned intervention.

6a-iii) Describe whether, how, and when qualitative feedback was obtained

Rating: 2/5

No structured qualitative interviews, focus groups, or open-ended feedback forms were conducted. Quantitative items captured the relevant outcomes; the limitations include the absence of richer qualitative data on the nature of distressing experiences.

6b) Any changes to trial outcomes after the trial commenced

No changes to trial outcomes were made after the trial commenced. The present analyses are pre-specified secondary analyses of an RCT, with the analytic plan preregistered on OSF (https://osf.io/94hks). Research Question 1 was preregistered as an exploratory analysis; Research Questions 2 and 3 were planned but not preregistered, and are clearly framed as such.

7a) How sample size was determined

From the Methods: "While no a priori power analysis was conducted for these secondary outcomes, the study had >80% power at $\alpha = .05$ to detect odds ratios of 1.55 or larger for distressing experience (base rate 4.1%) and 1.40 or larger for increased negative affect (base rate 7.1%) at the observed sample size." The parent trial was powered for its primary outcomes; the present manuscript is a secondary analysis.

7a-i) Describe whether and how expected attrition was taken into account when calculating the sample size

Rating: 2/5

Because this was a single-session online trial with a forced minimum-time-on-task constraint, no specific attrition adjustment was applied beyond the general expectation that some participants would not complete the survey. Observed attrition was low (2.5% missing for distressing experience; 2.8% missing for increased negative affect), and missingness analyses are reported in the Analysis Plan.

7b) When applicable, explanation of any interim analyses and stopping guidelines

Not applicable – no interim analyses or formal stopping rules were used. The trial proceeded to its planned sample size.

8a) Method used to generate the random allocation sequence

From the Methods: "participants were randomized via the randomizer function in the Qualtrics survey platform using a 4 x 2 x 2 factorial randomized trial design that crossed intervention type (mindfulness, self-compassion, gratitude, internet-as-usual) by intervention length (five minutes, ten minutes) and by speaker voice (male, female), with 14 unique conditions given that there was no speaker voice type in the control condition. The sample sizes in the control condition were double the size of the meditation conditions to retain balance necessary for optimal efficiency."

8b) Type of randomisation; details of any restriction (such as blocking and block size)

Allocation was implemented through the Qualtrics evenly-presented randomizer function, which assigns participants to conditions in approximately balanced proportions across the 14 unique conditions of the 4x2x2 factorial design. The control condition was double-weighted (double the size of each meditation condition) to retain balance for the factorial efficiency. No additional stratification or blocking beyond Prolific's representativeness sampling (age, sex, ethnicity) was applied.

9) Mechanism used to implement the random allocation sequence (such as concealment)

Random allocation was implemented automatically by the Qualtrics randomizer at the moment a participant completed baseline measures and was eligible to proceed to the intervention. Because randomization was fully automated and embedded in the survey flow, allocation was concealed from participants and from the research team until after assignment occurred (and, for participants, until they were presented with their assigned condition).

10) Who generated the random allocation sequence, who enrolled participants, and who assigned participants to interventions

Random allocation was generated and implemented by the Qualtrics survey platform. Enrollment was self-initiated by participants who clicked through from the Prolific Academic study listing to the Qualtrics consent page. No member of the research team manually enrolled participants or assigned them to conditions; assignment was fully automated.

11a-i) Specify who was blinded, and who wasn't

Rating: 4/5

Because participants received audio recordings with explicit instructions to engage in mindfulness, self-compassion, gratitude, or internet-as-usual, they could not be blinded to their condition – this is the typical situation in online behavioral RCTs. Outcome assessment was self-report, so participants were not blinded at outcome assessment. Data extraction from Qualtrics and statistical analyses were performed using condition labels rather than meaningful condition names where possible, but full blinding of the analyst was not implemented.

11a-ii) Whether participants knew which intervention was the "intervention of interest"

Rating: 3/5

Recruitment and consent materials described the study as a research project on online activities and well-being and did not single out a specific intervention as the "intervention of interest," so participants were not actively informed about which condition was the active vs comparator condition. However, participants who received a meditation recording could reasonably infer that meditation was the focus of the study.

11b) If relevant, description of the similarity of interventions

Not applicable in the placebo/sham sense. The active conditions (mindfulness, self-compassion, gratitude) and the active comparator (internet-as-usual) were intentionally distinct, with the comparator chosen as an ecologically valid real-world alternative to brief digital meditation rather than a sham. The three meditation conditions were matched on duration, audio recording format, and speaker voice options.

12a) Statistical methods used to compare groups for primary and secondary outcomes

Yes. The Analysis Plan section details the methods, including: logistic regression models for both adverse-experience outcomes; Bonferroni correction with statistical significance set at $P < .025$; Firth penalized logistic regression as a sensitivity analysis to address rare-event bias; and machine learning (elastic net regularization and random forest with nested ten-fold cross-validation, AUC as performance metric) for the prediction analyses.

12a-i) Imputation techniques to deal with attrition / missing values

Rating: 4/5

From the Methods: "Missing data on both outcomes was low (2.5% and 2.8%). We examined whether missingness was associated with key design variables. Missingness did not differ by intervention group, speaker voice, or baseline negative affect (ps > .133), but was higher in the ten-minute condition than the five-minute condition (3.5% vs 1.4% for distressing experience; 3.8% vs 1.8% for increased negative affect; Ps < .001). The magnitude of this length effect was comparable across active and control conditions (~2-fold increase in both), suggesting that elevated missingness in the ten-minute condition reflects task length rather than distress-related dropout. Given that missing data on both outcomes was less than 5%, which is commonly considered ignorable, we conducted complete case analyses." For the elastic net model, missing values among predictors were imputed (median for numeric, mode for nominal); for random forest, equivalent default handling was used.

12b) Methods for additional analyses, such as subgroup analyses and adjusted analyses

Additional analyses included interaction tests between intervention length and intervention type (active vs control) and between intervention length and specific meditation practices (mindfulness, self-compassion, gratitude); analogous interaction tests for speaker voice and meditation type; pairwise contrasts among the three active conditions using estimated marginal means; and the machine-learning prediction analysis (Research Question 3). All are described in the Analysis Plan.

X26-i) Comment on ethics committee approval

Rating: 5/5

From the Methods: "Study procedures were determined to be exempt from review by the Institutional Review Board at the University of Wisconsin-Madison and informed consent was obtained digitally from participants."

X26-ii) Outline informed consent procedures

Rating: 4/5

Informed consent was obtained online via Qualtrics before any study procedures. Participants were presented with a digital consent form describing the study procedures, time commitment (~25 minutes), compensation (3.2 USD / 2.5 GBP), data confidentiality, and their right to withdraw, and were required to indicate consent before proceeding to the baseline measures.

X26-iii) Safety and security procedures

Rating: 3/5

Data were collected through Qualtrics, which provides industry-standard encryption and secure storage. Participants were identified only by Prolific IDs and no directly identifying personal data were collected within the survey. The intervention itself involved brief audio recordings with no expected serious harm; participants were free to discontinue at any time. Adverse experiences were assessed as study outcomes (rather than monitored as safety endpoints), and no separate hotline was made available given the brief, single-session, low-risk nature of the intervention.

Results

13a) For each group, the numbers of participants randomly assigned, received intended treatment, and analysed for the primary outcome

Yes. From the Results: "A total of 5,260 individuals participated in the study, of which 155 participants (2.9%) were excluded because they failed the attention check ('I have been randomly selecting responses on this survey') and 56 participants (1.1%) were excluded due to incomplete responses. The

remaining 5,049 participants completed all pre-intervention measures and were randomly assigned to mindfulness (n = 1,262), self-compassion (n = 1,268), gratitude (n = 1,260), or control (n = 1,259; Figure 1)." Analytic Ns for each outcome are shown given the small amount of missing data on each outcome (2.5% and 2.8%).

13b) For each group, losses and exclusions after randomisation, together with reasons

Yes. The CONSORT flow diagram (Figure 1) reports randomization and analytic Ns by condition. From the Results: "Missing data for the two outcomes were minimal, with distressing experience missing for 2.5% of participants (n = 124) and increased negative affect missing for 2.8% of participants (n = 141)… missingness was higher in the ten-minute condition than the five-minute condition."

13b-i) Attrition diagram

Rating: 3/5

Because the trial was a single 5- or 10-minute session with a forced minimum time-on-task, sustained-engagement metrics over time (e.g., logins) are not meaningful. The CONSORT flow diagram (Figure 1) reports the relevant attrition information (assessment, exclusion at attention check, randomization, and missingness on outcomes by group).

14a) Dates defining the periods of recruitment and follow-up

From the Methods: "The data collection for this study occurred in 2023 between November and December." Because the intervention and outcome assessment were completed within a single ~25-minute session, no longer follow-up period applies.

14a-i) Indicate if critical "secular events" fell into the study period

Rating: 2/5

No major secular events specifically affecting Internet access, the Healthy Minds Program app, or Prolific/Qualtrics infrastructure occurred during the November–December 2023 data-collection window that we are aware of.

14b) Why the trial ended or was stopped (early)

The trial ended as planned once the target sample (representative of the US and UK adult populations with regards to age, sex, and ethnicity) had been recruited; it was not stopped early.

15) A table showing baseline demographic and clinical characteristics for each group

Partially. Table 1 reports baseline demographic and clinical characteristics for the full randomized sample rather than separately by condition.

15-i) Report demographics associated with digital divide issues

Rating: 4/5

Table 1 (and the Results text) report age, sex, ethnicity, sexual orientation, education, employment status, and prior meditation experience for the full randomized sample, all of which are relevant to digital-divide and ehealth-readiness considerations. Because participants were recruited via Prolific Academic, all participants were by definition active members of an online research panel and therefore had at least basic computer/internet literacy.

16) For each group, number of participants (denominator) included in each analysis and whether the analysis was by original assigned groups

Yes. Analyses were conducted on randomized participants in their assigned groups (intent-to-treat with respect to randomization), with complete-case exclusion of the small number of participants missing the relevant outcome (2.5% for distressing experience; 2.8% for increased negative affect). Group Ns are reported in Figure 1 and outcome-specific denominators are reflected in Tables 2 and 3 and the Supplemental Tables.

16-i) Report multiple "denominators" and provide definitions

Rating: 4/5

Multiple denominators are reported: total participants on Prolific entering the survey (5,260); participants excluded for failing attention check (155); participants excluded for incomplete responses (56); randomized analytic sample (5,049); and per-condition Ns: mindfulness (1,262), self-compassion (1,268), gratitude (1,260), control (1,259). Outcome-specific Ns reflect the additional 2.5% and 2.8% missing on each outcome, respectively. "Use" is defined as completing the full 5- or 10-minute audio recording (enforced by the survey).

16-ii) Primary analysis should be intent-to-treat

Rating: 4/5

Primary analyses were conducted in the randomized sample (5,049 participants) according to original group assignment, with complete-case exclusion only for participants missing the specific outcome (2.5% / 2.8%). Because the survey enforced a minimum time-on-task and exposure to the assigned intervention was effectively required for participation, randomized and "users" samples are essentially equivalent in this trial.

17a) For each primary and secondary outcome, results for each group, and the estimated effect size and its precision

Yes. Effect sizes (odds ratios) and 95% confidence intervals are reported throughout the Results and in Tables 2 and 3. Examples include: "no difference in the likelihood of reporting a distressing experience (odds ratio [OR] = 1.05, P = .763)"; "a significantly lower likelihood of increased negative affect relative those in the control condition (OR = 0.63, 95% CI [0.50, 0.80], P < .001)"; and "participants in the ten-minute condition had a significantly higher likelihood of reporting a distressing experience compared to those in the five-minute condition (OR = 1.42, 95% CI [1.07, 1.89], P = .015)." Discrimination performance for the prediction models is reported as AUC: "For distressing experience, our model achieved acceptable predictive performance with an AUC of .73… Performance was slightly lower for predicting increased negative affect (AUC = .67)."

17a-i) Presentation of process outcomes such as metrics of use and intensity of use

Rating: 3/5

Because the intervention was a single fixed-duration session with enforced minimum time-on-task, the only meaningful use metric is completion of the assigned 5- or 10-minute session, which is reflected in the analytic Ns. The manipulation check (in which participants identified the instruction they received post-intervention) provides an additional indicator of attention/exposure.

17b) For binary outcomes, presentation of both absolute and relative effect sizes is recommended

Both are reported. Absolute rates are reported in the Results: "Across the sample, 4.1% of participants reported having a distressing experience during the intervention and 7.1% of participants experienced an increase in negative affect from pre- to post-intervention." Relative effect sizes are reported as odds ratios with 95% CIs throughout the Results and in Tables 2 and 3.

18) Results of any other analyses performed, including subgroup analyses and adjusted analyses, distinguishing pre-specified from exploratory

Yes. The Analysis Plan and Results clearly distinguish preregistered exploratory analyses (Research Question 1, with the preregistration link https://osf.io/94hks) from additional planned-but-not-preregistered analyses (Research Questions 2 and 3). Sensitivity analyses using Firth penalized logistic regression are reported (Supplemental Tables 5 and 7), as are interaction analyses for intervention length × group type and length × meditation practice (Supplemental Table 6).

18-i) Subgroup analysis of comparing only users

Rating: 2/5

Because exposure to the assigned intervention was effectively enforced by the survey (minimum time-on-task) and observed missingness was very low, a separate "users-only" subgroup analysis would not differ meaningfully from the primary analyses and was therefore not conducted.

19) All important harms or unintended effects in each group

Yes. The primary outcomes of this manuscript are themselves operationalizations of harms / unintended effects (distressing experience and increased negative affect). Group-level rates and effect estimates are presented in the Results and Tables 2 and 3, with detailed item-level information in Supplemental Table 3. From the Results: "Across the sample, 4.1% of participants reported having a distressing experience during the intervention and 7.1% of participants experienced an increase in negative affect from pre- to post-intervention."

19-i) Include privacy breaches, technical problems

Rating: 3/5

No privacy breaches, technical problems, or other unexpected incidents were observed during the trial. The intervention also produced an unintended positive effect: participants randomized to a brief meditation intervention were less likely than control participants to experience a clinically relevant increase in negative affect (OR = 0.63, $P < .001$).

19-ii) Include qualitative feedback from participants or observations from staff/researchers

Rating: 2/5

Structured qualitative feedback was not collected; the limitations note this absence: future research with more detailed self-report checklists and qualitative interviews is recommended.

Discussion

22-i) Restate study questions and summarize the answers

Rating: 4/5

Yes. From the Discussion: "This study investigated adverse experiences in brief meditation practices using large, representative samples of the US and UK adult populations… Notably, the results showed that participants who were randomized to a brief meditation intervention were no more likely to report a distressing experience than those who were randomized to use the internet as usual. The results also showed that participants who were randomized to a brief meditation intervention were less likely to report clinically relevant increases in negative affect relative to using the internet as usual." Subsequent paragraphs summarize answers to Research Questions 2 and 3.

22-ii) Highlight unanswered new questions, suggest future research

Rating: 4/5

Yes. From the Discussion: "Future randomized controlled trials with more broadly representative samples, more objective and behavioral measures, more rigorous methodological safeguards, and a longer follow-up should aim to replicate these findings and externally validate data-driven approaches." Specific unanswered questions raised include the duration of effects, generalizability to populations with more meditation experience and to longer practices, and external validation of the predictive models.

20-i) Typical limitations in ehealth trials

Rating: 5/5

Yes. The Discussion includes a detailed limitations paragraph: "First, the measures of adverse experiences were self-reported, which has known biases (e.g., social desirability)… Second, given that different measurement approaches may yield systematically different prevalence estimates… it is possible that the prevalence estimates observed in the present study could represent lower bounds on the true rate of adverse experiences. Third, the sample was stratified to be representative of the US and UK national populations with respect to age, sex, and ethnicity, but it may not have been representative regarding other sociodemographic variables. Fourth, participants in the control condition were instructed to use the internet as they typically would, but the way they used the internet was neither systematically assessed nor objectively monitored… Fifth, the data-driven approach to identify predictors of adverse experiences was not externally validated… Sixth, the study only examined distressing experience during the intervention or immediate increases in negative affect post-intervention. The duration of any effects therefore remains unknown. Seventh, while we used an attention check to screen out careless responders, we did not implement additional approaches to detect careless response patterns…" Multiplicity is addressed via Bonferroni correction ($P < .025$) and rare-event bias via Firth penalized sensitivity analyses.

21-i) Generalizability to other populations

Rating: 4/5

From the Discussion: "the sample was stratified to be representative of the US and UK national populations with respect to age, sex, and ethnicity, but it may not have been representative regarding other sociodemographic variables… given the limited prior meditation experience reported by the sample, it is possible that these findings do not generalize to groups with more meditation experience." Findings should be considered "when meditation practices are recommended in cultural contexts shaped by structural inequities."

21-ii) Discuss differences between RCT and routine application setting

Rating: 3/5

The Discussion notes that the study tested a single brief, fixed-duration session with enforced time-on-task, which differs from routine app use where users self-select practices and durations: "It is also possible that these findings are specific to comparison between five- and ten-minute interventions and may not generalize to longer meditation durations." Routine app use also typically allows ad libitum dosing and includes additional onboarding and prompts that were not part of the trial.

Other Information

23) Registration number and name of trial registry

The trial's analytic plan was preregistered on the Open Science Framework (OSF): https://osf.io/94hks/overview. This is stated in the abstract ("Registration:

https://osf.io/94hks/overview") and in the Analysis Plan: "Our first research question was preregistered as an exploratory analysis (https://osf.io/94hks)."

24) Where the full trial protocol can be accessed

The preregistration, study materials, and analytic plan are available on the OSF project page: https://osf.io/94hks/overview. Supplemental Tables 1 and 2 in the manuscript provide the factorial design overview and the full list of survey items and questionnaires.

25) Sources of funding and other support, role of funders

From the manuscript Funding statement: "OS was supported by Ekhaga Foundation, Olle Engkvist Foundation, and Norrsken Mind. SBG was supported by the National Center for Complementary & Integrative Health award K23AT010879. MJH was supported by the National Institute of Mental Health award K01MH130752. ZJ was supported by the National Institute of Mental Health award T32MH018931. The content is solely the responsibility of the authors and does not necessarily represent the official views of the National Institutes of Health." Funders had no role in the design, conduct, analysis, or reporting of the study.

X27-i) State the relation of the study team towards the system being evaluated

Rating: 5/5

The meditation recordings used in this trial were drawn from the Healthy Minds Program app, developed by Healthy Minds Innovations, Inc., a non-profit affiliated with the Center for Healthy Minds at the University of Wisconsin–Madison. While several authors are affiliated with the Center for Healthy Minds Authors, no author receives royalties, equity, or other financial compensation from Healthy Minds Innovations or from the app, and the organization had no role in the design, conduct, analysis, or reporting of this study.

About the CONSORT EHEALTH Checklist

As a result of using this checklist, did you make changes in your manuscript?

Minor changes (or as appropriate, depending on which items you decide to add to the manuscript after reviewing your responses).

What were the most important changes you made as a result of using this checklist?

Clarified the closed/online nature of the trial and the level of human involvement (fully self-guided audio); explicitly noted that exposure to the assigned condition was enforced through a minimum time-on-task; expanded reporting of the rationale for the internet-as-usual comparator; and ensured that intervention provenance, accessibility, and the relationship between the study team and the Healthy Minds Program app were transparently disclosed.

How much time did you spend on going through the checklist including making changes in your manuscript

Approximately 2–3 hours.

As a result of using this checklist, do you think your manuscript has improved?

Yes.

Would you like to become involved in the CONSORT EHEALTH group?

No.

Any other comments or questions on CONSORT-EHEALTH

No.
