# Supplementary material for: Adverse Experiences in Brief Meditation Practices: Randomized Controlled Trial
Source: JMIR Ment Health. 2026 Sep 14;13:e95628. doi: 10.2196/95628 (PMC13693860; doi:10.2196/95628)
Supplement: Supplementary file 2 [file mental_v13i1e95628_app1.docx]

**Supplemental Tables**

**Supplemental Table 1. Factorial Design Overview**

| Intervention Type | Length | Speaker Voice |
| --- | --- | --- |
| Mindfulness | 5 min | Man |
| Mindfulness | 10 min | Man |
| Mindfulness | 5 min | Woman |
| Mindfulness | 10 min | Woman |
| Self-Compassion | 5 min | Man |
| Self-Compassion | 10 min | Man |
| Self-Compassion | 5 min | Woman |
| Self-Compassion | 10 min | Woman |
| Gratitude | 5 min | Man |
| Gratitude | 10 min | Man |
| Gratitude | 5 min | Woman |
| Gratitude | 10 min | Woman |
| Control | 5 min | None |
| Control | 10 min | None |

**Supplemental Table 2. Single survey items and questionnaires**

| **Single survey items** | |
| --- | --- |
| What is your age? | |
| Age | 18-100 |
| What is your gender? | |
| Gender | Female |
| Gender | Male |
| Gender | Non-binary gender |
| Gender | Other |
| Do you identify as transgender? | |
| Transgender | Yes |
| Transgender | No |
| What is your sexual orientation? | |
| Sexual orientation | Straight or heterosexual |
| Sexual orientation | Bisexual |
| Sexual orientation | Gay or lesbian |
| Sexual orientation | Other |
| What is your marital status? | |
| Marital status | Married |
| Marital status | Never married or single |
| Marital status | Living with a partner or in a committed, long-term relationship |
| Marital status | Separated |
| Marital status | Divorced |
| Marital status | Widowed |
| Marital status | Other |
| Which of the following best describes you? [US participants] | |
| Ethnicity | White or Caucasian |
| Ethnicity | Asian or Pacific Islander |
| Ethnicity | Black or African American |
| Ethnicity | Hispanic or Latino |
| Ethnicity | Native American or Alaskan Native |
| Ethnicity | Hispanic or Latino |
| Ethnicity | Other |
| Which of the following best describes you? [UK participants] | |
| Ethnicity | White |
| Ethnicity | Asian or Asian British |
| Ethnicity | Black, Black British, Caribbean or African |
| Ethnicity | Mixed or multiple ethnic groups |
| Ethnicity | Other |
| Do you have a bachelor's degree or higher? | |
| Educational attainment | Yes |
| Educational attainment | No |
| Have you ever experienced discrimination, been prevented from doing something, or been hassled or made to feel inferior because of your race, ethnicity, or color? | |
| Discrimination | Never |
| Discrimination | Once |
| Discrimination | Two or three times |
| Discrimination | Four or more times |
| How religious are you? | |
| Religious belief | Not at all religious |
| Religious belief | A little religious |
| Religious belief | Moderately religious |
| Religious belief | Quite religious |
| Religious belief | Very religious |
| Are you currently employed? | |
| Employment | Yes |
| Employment | No |
| Have you ever tried a meditation that involved paying attention to what is happening in the present moment? | |
| Ever tried mindfulness practice | Yes |
| Ever tried mindfulness practice | No |
| Please estimate your TOTAL LIFETIME number of hours of meditation practice that involved paying attention to what is happening in the present moment.* | |
| Hours of mindfulness practice | 0 |
| Hours of mindfulness practice | 0-10 |
| Hours of mindfulness practice | 11-100 |
| Hours of mindfulness practice | 101-500 |
| Hours of mindfulness practice | 501-1000 |
| Hours of mindfulness practice | 1001-5000 |
| Hours of mindfulness practice | 5000+ |
| Over the past 7 days, how many days did you engage in meditation practice that involved paying attention to what is happening in the present moment?* | |
| Past-week mindfulness practice | 0-7 |
| I personally have had distressing experiences as a result of meditation practice that involved paying attention to what is happening in the present moment. | |
| Adverse reaction to mindfulness practice | Never |
| Adverse reaction to mindfulness practice | Rarely |
| Adverse reaction to mindfulness practice | Occasionally |
| Adverse reaction to mindfulness practice | Regularly |
| Adverse reaction to mindfulness practice | Frequently |
| Have you ever tried a meditation that involved generating feelings of kindness? | |
| Ever tried kindness practice | Yes |
| Ever tried kindness practice | No |
| Please estimate your TOTAL LIFETIME number of hours of meditation practice that involved generating feelings of kindness.* | |
| Hours of kindness practice | 0 |
| Hours of kindness practice | 0-10 |
| Hours of kindness practice | 11-100 |
| Hours of kindness practice | 101-500 |
| Hours of kindness practice | 501-1000 |
| Hours of kindness practice | 1001-5000 |
| Hours of kindness practice | 5000+ |
| Over the past 7 days, how many days did you engage in meditation practice that involved generating feelings of kindness?* | |
| Past-week kindness practice | 0-7 |
| I personally have had distressing experiences as a result of meditation practice that involved generating feelings of kindness. | |
| Adverse reaction to kindness practice | Never |
| Adverse reaction to kindness practice | Rarely |
| Adverse reaction to kindness practice | Occasionally |
| Adverse reaction to kindness practice | Regularly |
| Adverse reaction to kindness practice | Frequently |
| Have you ever tried a meditation that involved generating feelings of gratitude or appreciation? | |
| Ever tried gratitude practice | Yes |
| Ever tried gratitude practice | No |
| Please estimate your TOTAL LIFETIME number of hours of meditation practice that involved generating feelings of gratitude or appreciation.* | |
| Hours of gratitude practice | 0 |
| Hours of gratitude practice | 0-10 |
| Hours of gratitude practice | 11-100 |
| Hours of gratitude practice | 101-500 |
| Hours of gratitude practice | 501-1000 |
| Hours of gratitude practice | 1001-5000 |
| Hours of gratitude practice | 5000+ |
| Over the past 7 days, how many days did you engage in meditation practice that involved generating feelings of gratitude or appreciation?* | |
| Past-week gratitude practice | 0-7 |
| I personally have had distressing experiences as a result of meditation practice that involved generating feelings of gratitude or appreciation. | |
| Adverse reaction to gratitude practice | Never |
| Adverse reaction to gratitude practice | Rarely |
| Adverse reaction to gratitude practice | Occasionally |
| Adverse reaction to gratitude practice | Regularly |
| Adverse reaction to gratitude practice | Frequently |
| Have you ever used the internet to access INFORMATION to support your mental health? | |
| Internet for mental health information | Yes |
| Internet for mental health information | No |
| Have you ever used the internet to access TREATMENTS or INTERVENTIONS (e.g., teletherapy, self-guided programs such as mindfulness training or cognitive behavioral therapy) to support your mental health? | |
| Internet for mental health treatments | Yes |
| Internet for mental health treatments | No |
| Have you ever used the internet to access FORUMS or SUPPORT GROUPS to support your mental health? | |
| Internet for mental health forums | Yes |
| Internet for mental health forums | No |
| Have you ever tried ONLINE / INTERNET-BASED cognitive behavioral therapy? | |
| iCBT | Yes |
| iCBT | No |
| Have you ever used social media to access INFORMATION to support your mental health? | |
| Social media for mental health information | Yes |
| Social media for mental health information | No |
| Have you ever used social media to access TREATMENTS OR INTERVENTIONS (e.g., teletherapy, self-guided programs such as mindfulness training or cognitive behavioral therapy) to support your mental health? | |
| Social media for mental health treatments | Yes |
| Social media for mental health treatments | No |
| Have you ever used social media to access FORUMS or SUPPORT GROUPS to support your mental health? | |
| Social media for mental health forums | Yes |
| Social media for mental health forums | No |
| Have you ever downloaded an app for your mental health? | |
| Downloaded app for mental health | Yes |
| Downloaded app for mental health | No |
| Have you ever used a smartphone app to access INFORMATION to support your mental health? | |
| App for mental health information | Yes |
| App for mental health information | No |
| Have you ever used a smartphone app to access TREATMENTS or INTERVENTIONS (e.g., teletherapy, self-guided programs such as mindfulness training or cognitive behavioral therapy) to support your mental health? | |
| App for mental health treatments | Yes |
| App for mental health treatments | No |
| Have you ever used ChatGPT or similar software based on artificial intelligence to support your mental health? | |
| AI-based software for mental health | Yes |
| AI-based software for mental health | No |
| Do you own a smartphone? | |
| Smartphone ownership | Yes |
| Smartphone ownership | No |
| Do you have regular access to the internet? | |
| Regular access to internet | Yes |
| Regular access to internet | No |
| Did you have a distressing experience during the task you just completed? | |
| Distressing experience | Yes |
| Distressing experience | No |
| **Questionnaires** | |
| Ten-Item Personality Inventory | |
| Well-Being Growth Mindset, adapted from the Growth Mindset Scale of intelligence | |
| Determinants of Meditation Practice Inventory-Revised | |
| Short Version Ruminative Response Style | |
| NIH Toolbox Loneliness | |
| Perceived Stress Scale – 4 items version | |
| PROMIS Anxiety | |
| PROMIS Depression | |
| Five Facets of Mindfulness Questionnaire | |
| Self-Compassion Scale – Short Form | |
| Gratitude Questionnaire | |
| Positive and Negative Affect Schedule | |

*Note.* Questionnaires with past-tense questions (e.g., in the past week) were adjusted to ask about the past two days.

**Supplemental Table 3. Adverse experiences by factorial design**

| Type | Condition | N | Distressing Experience, n (%) | Increased Negative Affect, n (%) |
| --- | --- | --- | --- | --- |
| Intervention | Control | 1259 | 49 (4.0) | 117 (9.6) |
| Intervention | Gratitude | 1260 | 59 (4.8) | 83 (6.8) |
| Intervention | Self-Compassion | 1268 | 46 (3.7) | 72 (5.9) |
| Intervention | Mindful | 1262 | 50 (4.0) | 76 (6.2) |
| Length | Five Minute | 2525 | 86 (3.5) | 176 (7.1) |
| Length | Ten Minute | 2524 | 118 (4.8) | 172 (7.1) |
| Speaker Voice | Woman | 1896 | 80 (4.3) | 124 (6.7) |
| Speaker Voice | Man | 1894 | 75 (4.1) | 107 (5.8) |

**Supplemental Table 4. Missingness by each of the factorial conditions**

| Type | Condition | N | Distressing Experience, n (%) | Increased Negative Affect, n (%) |
| --- | --- | --- | --- | --- |
| Intervention | Control | 1259 | 32 (2.5) | 34 (2.7) |
| Intervention | Gratitude | 1260 | 26 (2.1) | 31 (2.5) |
| Intervention | Self-Compassion | 1268 | 39 (3.1) | 47 (3.7) |
| Intervention | Mindful | 1262 | 27 (2.1) | 29 (2.3) |
| Length | Five Minute | 2525 | 36 (1.4) | 45 (1.8) |
| Length | Ten Minute | 2524 | 88 (3.5) | 96 (3.8) |
| Speaker Voice | Woman | 1896 | 39 (2.1) | 46 (2.4) |
| Speaker Voice | Man | 1894 | 53 (2.8) | 61 (3.2) |

**Supplemental Table 5. Examining the likelihood of adverse experiences by group assignment using Firth logistic regression**

| Model | Outcome | Comparison | OR | 95% CI | *z* | *P* |
| --- | --- | --- | --- | --- | --- | --- |
| Active vs control | Distressing experience | Active vs Control | 1.04 | [0.76, 1.46] | 0.26 | .793 |
| Active vs control | Increased negative affect | Active vs Control | 0.63 | [0.5, 0.8] | -3.87 | <.001 |
| Among active | Distressing experience | Self-compassion vs Gratitude | 0.78 | [0.52, 1.15] | -1.27 | .205 |
| Among active | Distressing experience | Mindfulness vs Gratitude | 0.84 | [0.57, 1.23] | -0.88 | .378 |
| Among active | Increased negative affect | Self-compassion vs Gratitude | 0.87 | [0.62, 1.2] | -0.87 | .386 |
| Among active | Increased negative affect | Mindfulness vs Gratitude | 0.91 | [0.66, 1.25] | -0.59 | .553 |

*Note*. OR = odd ratio; CI = confidence interval. Groups listed second in the comparison column were coded as the reference group.

**Supplemental Table 6. Examining the interactions between group assignment and intervention length and speaker voice to predict adverse experiences**

| Model | Outcome | Model Type | Comparison | OR | 95% CI | t | df | *P* |
| --- | --- | --- | --- | --- | --- | --- | --- | --- |
| Length x Active | Distressing experience | Interaction | Ten Min x Active | 1.31 | [0.67, 2.53] | 0.80 | 4921 | .426 |
| Length x Group | Distressing experience | Interaction | Ten Min x Self-compassion | 0.54 | [0.24, 1.20] | -1.50 | 3692 | .134 |
| Length x Group | Distressing experience | Interaction | Ten Min x Mindfulness | 0.88 | [0.4, 1.97] | -0.31 | 3692 | .760 |
| Length x Active | Increased negative affect | Interaction | Ten Min x Active | 1.75 | [1.09, 2.8] | 2.32 | 4904 | .020 |
| Length x Group | Increased negative affect | Interaction | Ten Min x Self-compassion | 0.82 | [0.42, 1.59] | -0.58 | 3677 | .564 |
| Length x Group | Increased negative affect | Interaction | Ten Min x Mindfulness | 0.51 | [0.26, 0.97] | -2.03 | 3677 | .043 |
| Voice x Group | Distressing experience | Interaction | Man Voice x Self-compassion | 0.74 | [0.33, 1.63] | -0.75 | 3692 | .453 |
| Voice x Group | Distressing experience | Interaction | Man Voice x Mindfulness | 1.23 | [0.57, 2.67] | 0.52 | 3692 | .604 |
| Voice x Group | Increased negative affect | Interaction | Man Voice x Self-compassion | 1.20 | [0.62, 2.32] | 0.54 | 3677 | .587 |
| Voice x Group | Increased negative affect | Interaction | Man Voice x Mindfulness | 1.49 | [0.78, 2.86] | 1.21 | 3677 | .228 |

*Note*. OR = odd ratio; CI = confidence interval. df = degrees of freedom. Groups listed in the comparison column were coded as the focal group (1), with the unlisted groups (e.g., Gratitude) serving as the reference (0; e.g., Ten Min = 1, Five Min = 0).

**Supplemental Table 7. Examining the likelihood of adverse experiences by intervention length and speaker voice using Firth logistic regression**

| Model | Outcome | Model Type | Comparison | OR | 95% CI | z | *P* |
| --- | --- | --- | --- | --- | --- | --- | --- |
| Intervention length | Distressing experience | Main effect | Ten vs Five Minutes | 1.42 | [1.07, 1.89] | 0.35 | .015 |
| Intervention length | Increased negative affect | Main effect | Ten vs Five Minutes | 1.00 | [0.8, 1.24] | 0.00 | .987 |
| Speaker voice | Distressing experience | Main effect | Man vs Woman Voice | 0.94 | [0.68, 1.30] | -0.06 | .723 |
| Speaker voice | Increased negative affect | Main effect | Man vs Woman Voice | 0.86 | [0.66, 1.13] | -0.15 | .280 |

*Note*. OR = odd ratio; CI = confidence interval. Groups listed second in the comparison column were coded as the reference group.

**Supplemental Table 8. Standardized regression coefficients**

| Variable | *β* (distressing experience) | *β* (increased negative affect) |
| --- | --- | --- |
| Negative affect | 0.12 | 0.00 |
| Discrimination experiences | 0.10 | 0.00 |
| Transgender identity | 0.09 | 0.00 |
| Anxiety | 0.09 | 0.00 |
| Depression | 0.09 | 0.11 |
| Growth mindset | -0.07 | 0.00 |
| Gratitude | -0.06 | -0.22 |
| Agreeableness | -0.06 | -0.08 |
| Openness | 0.06 | 0.01 |
| Race: Black | 0.05 | 0.00 |
| Female gender | -0.05 | -0.11 |
| Mindfulness: Nonjudge | -0.05 | -0.19 |
| Mindfulness: Observe | 0.05 | 0.00 |
| DMPI: Cultural barriers | 0.05 | 0.02 |
| DMPI: Pragmatic barriers | 0.05 | 0.03 |
| Emotional stability | -0.05 | -0.07 |
| Stress | 0.05 | 0.00 |
| Internet mental health forums | 0.04 | 0.00 |
| DMPI: Perceived benefit | -0.04 | 0.00 |
| Conscientiousness | -0.04 | -0.10 |
| Race: Other | 0.04 | 0.00 |
| ChatGPT for mental health | 0.04 | 0.00 |
| Internet-based CBT | 0.04 | 0.10 |
| Ever tried kindness practice | 0.04 | 0.03 |
| Positive affect | 0.04 | 0.07 |
| Self-compassion: Common humanity | -0.04 | -0.02 |
| Extraversion | 0.04 | 0.05 |
| Rumination: Reflection | -0.04 | -0.05 |
| Religiosity | 0.04 | 0.10 |
| Kindness practice lifetime hours | 0.04 | 0.00 |
| Currently employed | -0.04 | 0.05 |
| Loneliness | 0.03 | 0.00 |
| Mindfulness: Awareness | -0.03 | 0.00 |
| Ever tried mindfulness practice | -0.03 | 0.06 |
| Self-compassion: Self-judgment | -0.03 | 0.09 |
| Internet access | -0.03 | 0.00 |
| Gratitude group | 0.03 | -0.07 |
| College education | -0.03 | 0.07 |
| In relationship | -0.03 | 0.00 |
| Mindfulness practice lifetime hours | -0.03 | 0.00 |
| Mental health app | -0.03 | -0.04 |
| Social media mental health forums | 0.03 | 0.00 |
| DMPI: Knowledge barriers | -0.03 | -0.09 |
| Kindness practice days per week | 0.02 | 0.00 |
| Mindfulness: Nonreact | 0.02 | 0.00 |
| Internet mental health treatment | -0.02 | 0.00 |
| Heterosexual orientation | -0.02 | -0.09 |
| Self-compassion: Total | -0.02 | 0.00 |
| Phone mental health treatment | 0.02 | 0.00 |
| Self-compassion group | -0.02 | -0.10 |
| Internet mental health information | -0.02 | 0.00 |
| DMPI: Total | 0.02 | 0.00 |
| Social media mental health treatment | -0.01 | 0.00 |
| Mindfulness: Describe | 0.01 | 0.00 |
| Race: Asian | 0.01 | 0.00 |
| Rumination: Brooding | 0.01 | 0.00 |
| Ever tried gratitude practice | -0.01 | 0.00 |
| Self-compassion: Mindfulness | -0.01 | 0.00 |
| Phone mental health information | 0.01 | -0.05 |
| Mindfulness group | -0.01 | -0.11 |
| Self-compassion: Kindness | -0.01 | -0.04 |
| Gratitude practice days per week | 0.01 | 0.00 |
| Gratitude practice lifetime hours | -0.01 | -0.01 |
| Social media mental health information | 0.01 | 0.00 |
| Race: Mixed | 0.00 | 0.00 |
| Mindfulness: Total | 0.00 | 0.00 |
| Age | 0.00 | 0.00 |
| Self-compassion: Isolation | 0.00 | 0.00 |
| Self-compassion: Over-identification | 0.00 | 0.00 |
| Mindfulness practice days per week | 0.00 | 0.00 |

*Note. β* = standardized beta values. DMPI = Determinants of Meditation Practice Inventory-Revised. CBT = Cognitive Behavioral Therapy.
